# Supplementary material for: Rising together: exploring sourdough fermentation diversity through co-design in the HealthFerm citizen science initiative
Source: NPJ Sci Food. 2026 Jan 27;10:76. doi: 10.1038/s41538-026-00725-7 (PMC12948946; doi:10.1038/s41538-026-00725-7)
Supplement: Supplementary file 1 — Supplementary data [file 41538_2026_725_MOESM1_ESM.pdf]

Supplementary information

Rising together: Exploring sourdough fermentation diversity through co-design in the HealthFerm citizen science initiative

Subtitle: Sourdough starter characteristics, baker demographics, and fermentation practices across Europe

Annina Meyer*[,1], Thomas Gettemans*[,2], Jan Patrick Tan*[,3], Fabio Tuccillo[4], Chiara Viretto[5], Iulia-Roxana Angelescu[6], Yamina De Bondt[7], Michelle Neugebauer[1], Ali Zein Alabiden Tlais[5], Fabio Cavelti[1], Luc De Vuyst[2], Marco Gobbetti[5], Christophe M. Courtin[7], Medana Zamfir[6], Rossana Coda[4], Laura Nyström[3], Stefan Weckx[2], Nicholas A. Bokulich[1#]

*These authors contributed equally

Affiliations

1. Laboratory of Food Systems Biotechnology, Department of Health Sciences and Technology, ETH Zurich, Schmelzbergstrasse 7, 8092 Zurich, Switzerland
2. Research Group of Industrial Microbiology and Food Biotechnology (IMDO), Faculty of Sciences and Bioengineering Sciences, Vrije Universiteit Brussel (VUB), Pleinlaan 2, B-1050 Brussels, Belgium
3. Laboratory of Food Biochemistry, Department of Health Sciences and Technology, ETH Zurich, Schmelzbergstrasse 9, 8092 Zurich, Switzerland
4. Department of Food and Nutrition, University of Helsinki, Agnes Sjöbergin katu 2, 00014 Helsinki, Finland
5. Faculty of Agricultural, Environmental and Food Sciences, Free University of Bolzano-Bozen, 39100 Bolzano, Italy
6. Institute of Biology Bucharest of the Romanian Academy, 296 Splaiul Independentei, Bucharest, Romania
7. Laboratory of Food Chemistry and Biochemistry and Leuven Food Science and Nutrition Research Centre (LFoRCe), KU Leuven, Kasteelpark Arenberg 20, B-3001 Leuven, Belgium

#Correspondence
nicholas.bokulich@hest.ethz.ch

Supplementary figures

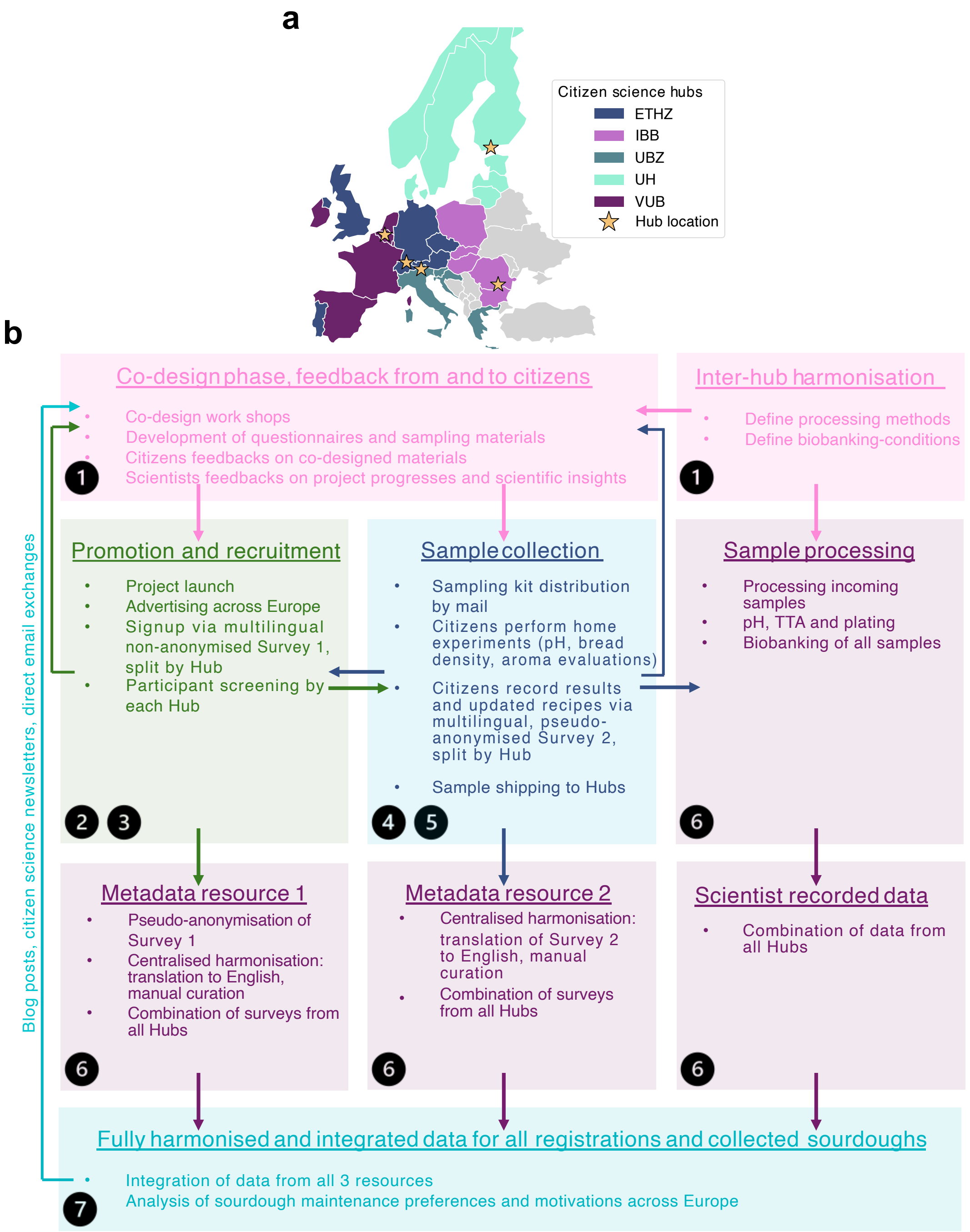

Supplementary Figure 1. Methodological framework and project stages of the co-design-driven HealthFerm sourdough citizen science initiative. a, Participating countries and laboratory hubs: ETHZ: ETH Zurich, Switzerland; IBB: Institute of Biology Bucharest, Romania; UBZ: Free University of Bozen-Bolzano, Italy; UH: University of Helsinki, Finland; VUB: Vrije Universiteit Brussel, Belgium. **b,** Schematic outlining the stepwise approach underlying the initiative, including: (1) the co-design phase, involving bidirectional feedback between citizens and scientists; (2–3) advertisement and participant recruitment via Survey 1; (4–5) sample collection and home-based experimental activities, home experiment result reporting via Survey 2; (6) unified sample processing across hubs and centralised harmonisation of survey data; and (7) integrated survey data analysis. Communication and engagement channels (e.g., blog posts on the HealthFerm webpage, newsletters, and direct exchanges) supported participant involvement throughout the process.

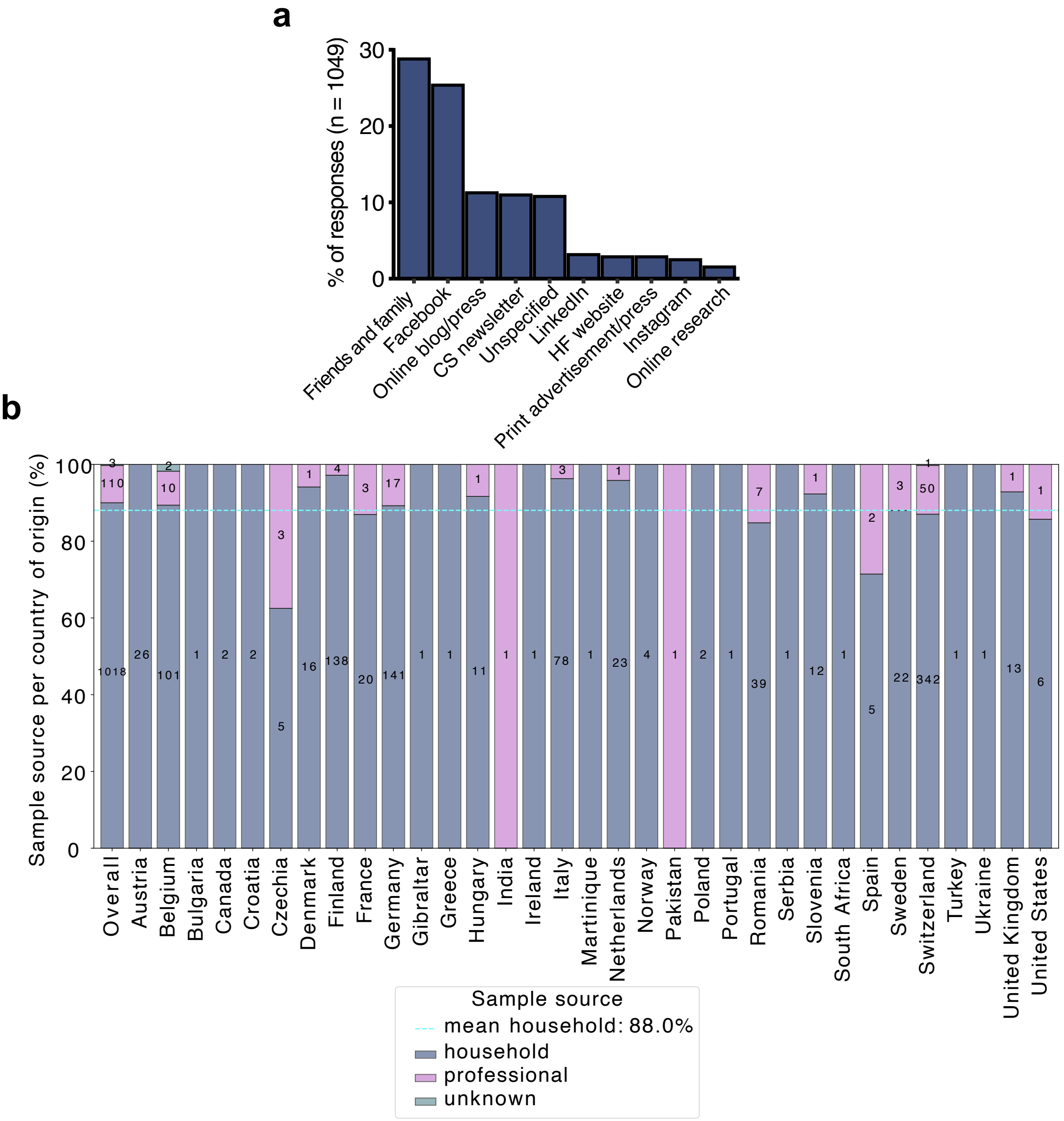

Supplementary Figure 2. Advertisement platforms and signup distributions across countries. a, Channels through which participants got in contact with the project team. **b**, Distribution of household and bakery samples across countries. The mean percentage of household samples across all countries is highlighted. Numbers within bars indicate absolute response counts per category.

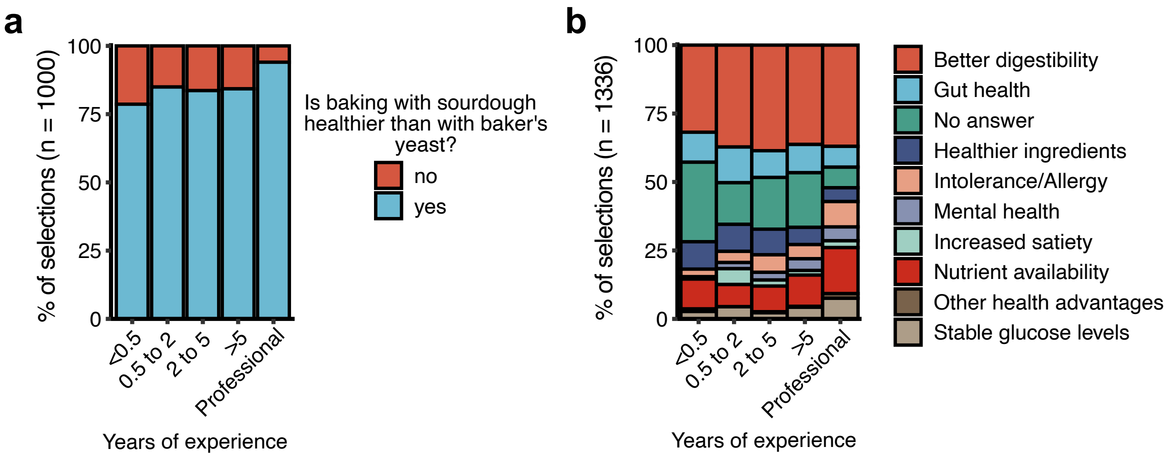

Supplementary Figure 3. Perceived health benefits across skill levels. **a**, Perception of sourdough bread being healthier than yeast-leavened bread, stratified by self-rated sourdough baking skill levels of all respondents. **b**, Perceived health benefits as specified by participants, stratified by self-rated sourdough baking skill levels of all respondents.

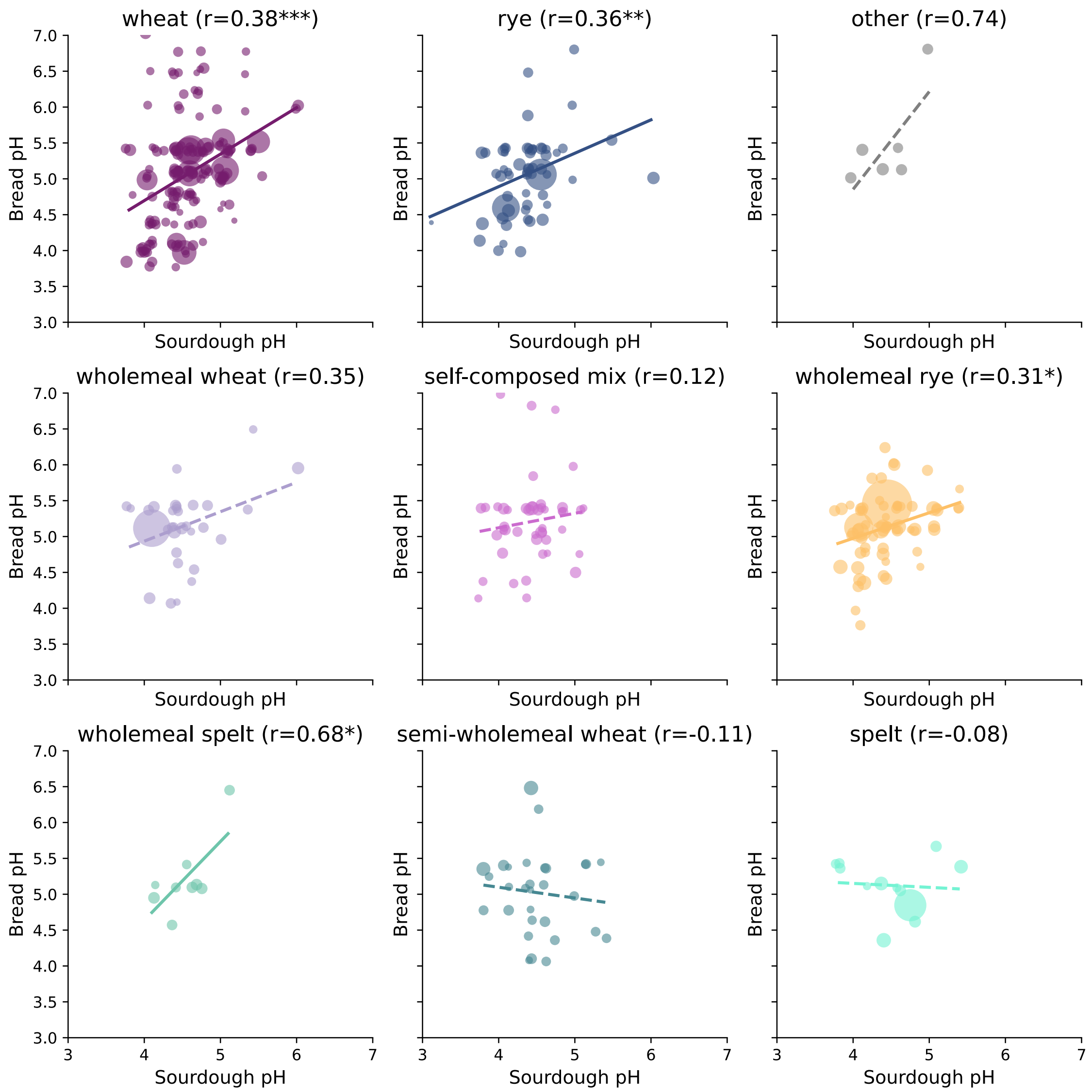

Supplementary Figure 4. Sourdough and bread pH correlations split by flour type. Pearson correlations between sourdough pH and sourdough bread pH as determined in home experiments, split by flour type. Significance of the correlation is marked according to p-values: *<0.05; **<0.01, ***<0.001. Bubble sizes are proportional to the bread density assessed by citizens (bigg bubble size: high bread density; small bubble size: low bread density).

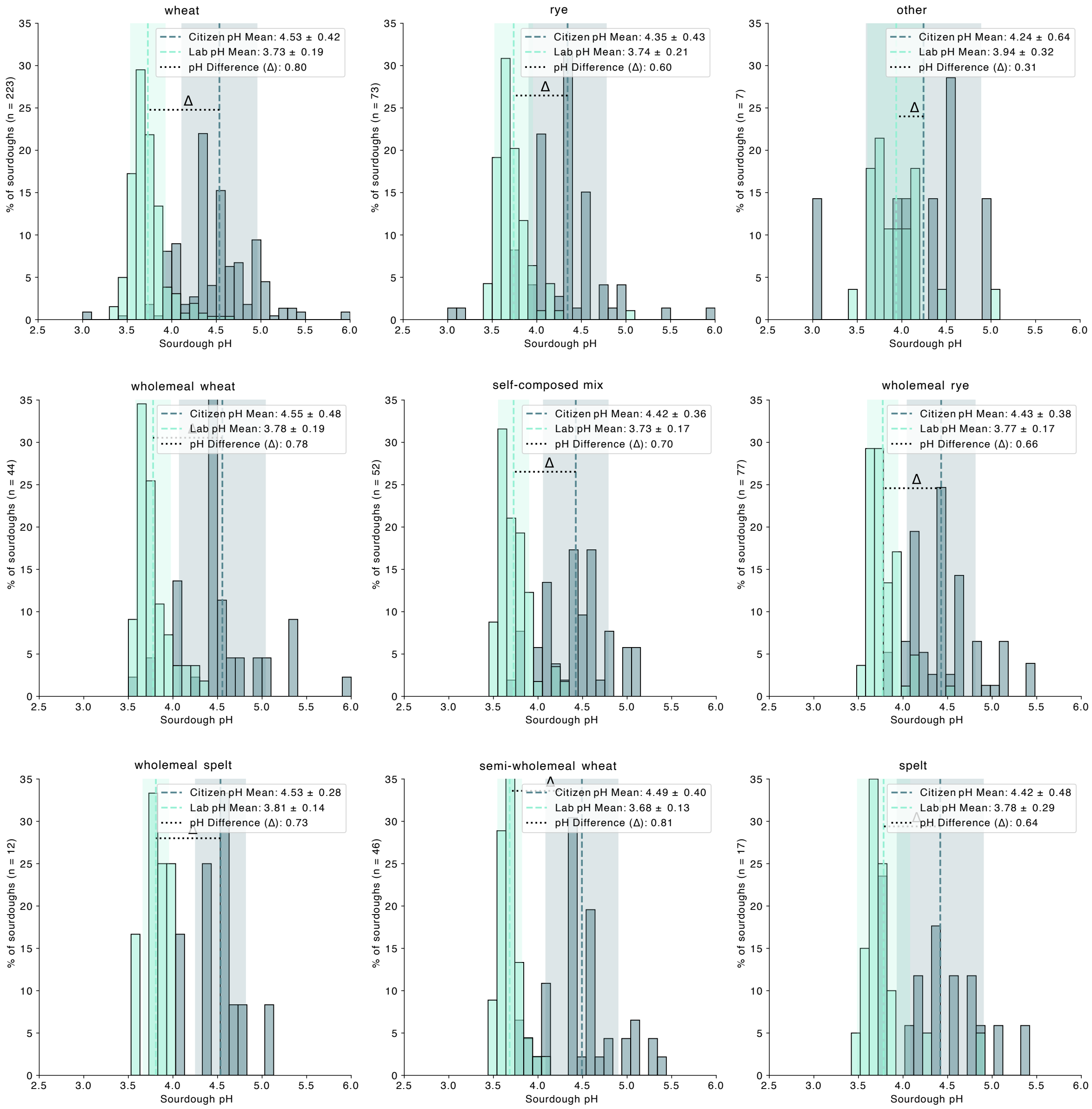

Supplementary Figure 5. Distributions of at-home measured and in the laboratory measured sourdough pH. pH shifts by sourdough flour type, comparing participant-measured pH at home with laboratory pH measurements after sample shipping.

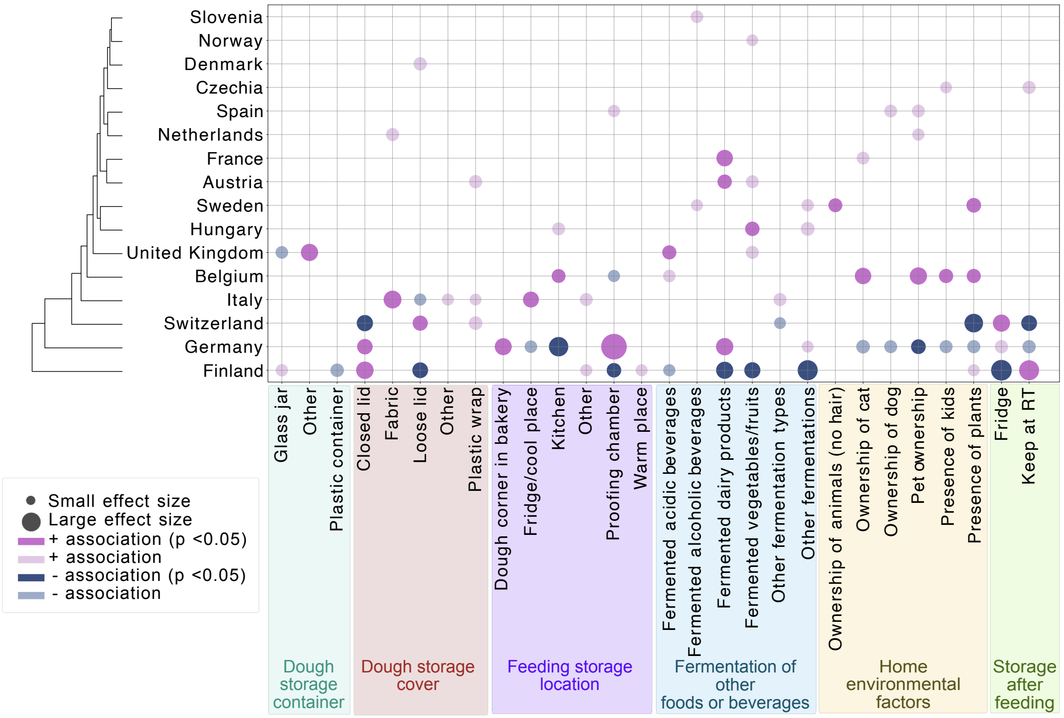

Supplementary Figure 6. Sourdough maintenance practices and home environments among participants across European countries. Bubble plot clustering countries based on similarities in sourdough maintenance practices and home environments. This includes dough storage devices and covers, feeding locations, whether participants ferment other foods or beverages, environmental factors, and where they store their sourdough after feeding. Only countries and subcategories with at least one significant Chi-square association across tested variables are displayed. Associations are colour-coded based on their statistical significance (darker shade: BH-FDR corrected p-value < 0.05; lighter shade: uncorrected p-value < 0.05) and whether they show positive or negative enrichment within a given country, non-significant associations are not displayed. Countries are hierarchically clustered based on effect size (Cramér's V) across all features, including both significant and non-significant enrichments, and bubble size corresponds to effect size (Cramér's V).

Supplementary Tables

Supplementary Table 1. Contents of the sampling kit and the purpose of each item.

**Items**	**Purpose**
Instruction booklet	Provides an introduction to the project, graphical instructions for the sampling procedure and the at-home experiments along with a QR-code directing to the result questionnaire (deposited on Zenodo; https://doi.org/10.5281/zenodo.15366921)
Sampling container	A sterile container to avoid microbial contamination from participant-provided containers.
Plastic freezer bag	Provides a second barrier in case of sample leakage during shipment.
pH strips and flavour wheels	Required items for performing the at-home experiments.
Prepaid return shipment label	Facilitates sample shipment and covers any costs from the participant.
Project-branded pen and sticker	Souvenir for participation.

Supplementary Table 2. Results of Chi-square enrichment tests for advertisement channels by country (corresponding to Figure 2E). p-values were corrected using the Benjamini–Hochberg false discovery rate (BH-FDR). Indicator codes: PE = positive enrichment, NE = negative enrichment, M = multiple indicators, U = unique indicator.

Category	*Cluster*	*Chi2 Statistic*	*Cramér's V*	*Category count*	*Cluster Total*	*Category Total*	*Raw p-value*	*Corrected p-value*	*Enrichment Score*	*Interpretation*	*Indicator*
bakery	Belgium	3.2207	0.0551	6	90	32	0.0727	0.1309	2.2104	PE	M
bakery	Switzerland	5.8903	0.0745	18	367	32	0.0152	0.0433	1.6262	PE	M
citizen science or uni newsletter	Germany	0.0473	0.0067	6	149	50	0.8278	0.9511	0.8545	NE	M
citizen science or uni newsletter	Belgium	1.3794	0.0361	7	90	50	0.2402	0.3706	1.6504	PE	M
citizen science or uni newsletter	Austria	4.5433	0.0654	4	26	50	0.0330	0.0744	3.2646	PE	M
citizen science or uni newsletter	Switzerland	4.8158	0.0674	25	367	50	0.0282	0.0662	1.4455	PE	M
citizen science or uni newsletter	Slovenia	6.1778	0.0763	3	13	50	0.0129	0.0411	4.8969	PE	M
direct email	Germany	4.9603	0.0684	14	149	56	0.0259	0.0637	1.7802	PE	M
direct email	Belgium	0.1365	0.0113	6	90	56	0.7118	0.8356	1.2631	PE	M
direct email	Romania	4.2898	0.0636	6	46	56	0.0383	0.0828	2.4713	PE	M
direct email	Switzerland	0.8161	0.0277	23	367	56	0.3663	0.5346	1.1874	PE	M
Facebook	Finland	360.2175	0.5827	126	139	267	0.0000	0.0000	3.6021	PE	M
Facebook	Sweden	11.3041	0.1032	14	25	267	0.0008	0.0032	2.2253	PE	M
Facebook	Germany	39.8325	0.1938	6	149	267	0.0000	0.0000	0.1600	NE	M
Facebook	Netherlands	12.5994	0.1090	14	24	267	0.0004	0.0017	2.3180	PE	M
Facebook	Belgium	0.6391	0.0245	19	90	267	0.4240	0.5871	0.8389	NE	M
Facebook	France	17.9115	0.1299	15	23	267	0.0000	0.0001	2.5916	PE	M
Facebook	Austria	0.2277	0.0146	5	26	267	0.6332	0.7787	0.7642	NE	M
Facebook	Romania	2.9258	0.0525	17	46	267	0.0872	0.1518	1.4686	PE	M
Facebook	Switzerland	117.4117	0.3327	19	367	267	0.0000	0.0000	0.2057	NE	M
Facebook	Italy	3.7341	0.0593	12	78	267	0.0533	0.0993	0.6114	NE	M
Facebook	Slovenia	0.0216	0.0045	4	13	267	0.8831	0.9873	1.2227	PE	M

Facebook	Spain	0.4164	0.0198	3	7	267	0.5187	0.7003	1.7030	PE	M
friends and family	Finland	28.9857	0.1653	9	139	269	0.0000	0.0000	0.2554	NE	M
friends and family	Sweden	0.7315	0.0263	4	25	269	0.3924	0.5576	0.6311	NE	M
friends and family	Denmark	0.2073	0.0140	3	17	269	0.6489	0.7787	0.6960	NE	M
friends and family	United Kingdom	5.9690	0.0750	8	14	269	0.0146	0.0433	2.2539	PE	M
friends and family	Germany	5.2462	0.0703	26	149	269	0.0220	0.0594	0.6883	NE	M
friends and family	Belgium	0.3447	0.0180	20	90	269	0.5571	0.7338	0.8765	NE	M
friends and family	Czechia	7.8586	0.0861	5	6	269	0.0051	0.0171	3.2869	PE	M
friends and family	Romania	4.0915	0.0621	18	46	269	0.0431	0.0875	1.5434	PE	M
friends and family	Switzerland	10.1301	0.0977	115	367	269	0.0015	0.0056	1.2359	PE	M
friends and family	Hungary	18.5717	0.1323	10	12	269	0.0000	0.0001	3.2869	PE	M
friends and family	Italy	2.3959	0.0475	26	78	269	0.1217	0.1991	1.3147	PE	M
friends and family	Slovenia	0.0171	0.0040	4	13	269	0.8959	0.9873	1.2136	PE	M
HealthFerm website	Belgium	5.1177	0.0695	7	90	34	0.0237	0.0609	2.4271	PE	M
HealthFerm website	Austria	17.0908	0.1269	5	26	34	0.0000	0.0002	6.0011	PE	M
HealthFerm website	Switzerland	1.8779	0.0421	16	367	34	0.1706	0.2709	1.3605	PE	M
Instagram	Switzerland	3.7585	0.0595	15	367	28	0.0525	0.0993	1.5488	PE	U
LinkedIn	Denmark	191.0073	0.4243	11	17	34	0.0000	0.0000	20.1920	PE	M
LinkedIn	Switzerland	0.2134	0.0142	10	367	34	0.6441	0.7787	0.8503	NE	M
online research	Belgium	8.0738	0.0872	5	90	16	0.0045	0.0162	3.6840	PE	M
online research	Switzerland	1.0836	0.0320	8	367	16	0.2979	0.4468	1.4455	PE	M
other	Switzerland	0.2888	0.0165	18	367	59	0.5910	0.7598	0.8820	NE	M
other	Italy	224.0940	0.4596	34	78	59	0.0000	0.0000	7.8388	PE	M
press	Sweden	2.7746	0.0511	6	25	122	0.0958	0.1616	2.0872	PE	M
press	Belgium	0.0027	0.0016	11	90	122	0.9583	1.0000	1.0629	PE	M
press	Switzerland	129.7522	0.3497	99	367	122	0.0000	0.0000	2.3460	PE	M
press	Italy	4.0670	0.0619	3	78	122	0.0437	0.0875	0.3345	NE	M
sourdough blog	Germany	577.7783	0.7379	91	149	94	0.0000	0.0000	6.8935	PE	U

Supplementary Table 3. Chi-square enrichment test results for baking motivations across flour types used (related to Figure 4C). p-values were corrected using the Benjamini–Hochberg false discovery rate (BH-FDR). Indicator codes: PE = positive enrichment, NE = negative enrichment, M = multiple indicators, U = unique indicator.

Feature	*Category*	*Cluster*	*Chi2 Statistic*	*Cramér's V*	*Category count*	*Cluster Total*	*Category Total*	*Raw p-value*	*Corrected p-value*	*Enrichment Score*	*Interpretation*	*Indicator*
Baking Motivation: Enjoy Baking	yes	endosperm	0.5807	0.0227	445	638	800	0.4460	0.6918	0.9861	NE	M
Baking Motivation: Enjoy Baking	yes	mix	6.3947	0.0752	116	145	800	0.0114	0.0870	1.1310	PE	M
Baking Motivation: Enjoy Baking	yes	organic: no	0.0313	0.0053	309	432	800	0.8596	0.8596	0.9942	NE	M
Baking Motivation: Enjoy Baking	yes	other	4.8249	0.0664	116	145	788	0.0281	0.1247	1.1107	PE	M
Baking Motivation: Enjoy Baking	yes	other	17.1397	0.1231	10	29	800	0.0000	0.0007	0.4875	NE	M
Baking Motivation: Enjoy Baking	yes	rye	0.0115	0.0032	212	296	788	0.9147	0.9628	0.9943	NE	M
Baking Motivation: Enjoy Baking	yes	spelt	5.3434	0.0699	29	51	788	0.0208	0.1234	0.7894	NE	M
Baking Motivation: Enjoy Baking	yes	wheat	0.0821	0.0087	431	602	788	0.7745	0.9628	0.9940	NE	M
Baking Motivation: Enjoy Baking	yes	wholemeal	0.1724	0.0123	229	319	800	0.6780	0.8437	1.0149	PE	M
Baking Motivation: Enjoy Baking	yes	organic: yes	0.0313	0.0053	491	680	800	0.8596	0.8596	1.0037	PE	M
Baking Motivation: Health	yes	endosperm	1.1958	0.0325	317	638	579	0.2742	0.5893	0.9706	NE	M
Baking Motivation: Health	yes	mix	0.0948	0.0092	72	145	579	0.7581	0.8730	0.9699	NE	M
Baking Motivation: Health	yes	organic: no	4.0970	0.0607	208	432	579	0.0430	0.2148	0.9247	NE	M
Baking Motivation: Health	yes	other	0.1812	0.0129	72	145	565	0.6703	0.8938	0.9615	NE	M
Baking Motivation: Health	yes	other	1.5859	0.0374	11	29	579	0.2079	0.5554	0.7409	NE	M
Baking Motivation: Health	yes	rye	10.3556	0.0973	177	296	565	0.0013	0.0129	1.1578	PE	M
Baking Motivation: Health	yes	spelt	0.2786	0.0160	24	51	565	0.5976	0.8333	0.9112	NE	M
Baking Motivation: Health	yes	wheat	5.0102	0.0677	292	602	565	0.0252	0.1247	0.9392	NE	M
Baking Motivation: Health	yes	wholemeal	4.0334	0.0597	179	319	579	0.0446	0.2825	1.0961	PE	M

Baking Motivation: Health	yes	organic: yes	4.0970	0.0607	371	680	579	0.0430	0.2148	1.0478	PE	M
Baking Motivation: Lower Cost	yes	endosper m	0.4422	0.0198	87	638	162	0.5061	0.7439	0.9520	NE	M
Baking Motivation: Lower Cost	yes	mix	0.6890	0.0247	17	145	162	0.4065	0.6866	0.8185	NE	M
Baking Motivation: Lower Cost	yes	organic: no	0.2001	0.0134	66	432	162	0.6547	0.8510	1.0487	PE	M
Baking Motivation: Lower Cost	yes	other	0.8176	0.0273	17	145	159	0.3659	0.6418	0.8067	NE	M
Baking Motivation: Lower Cost	yes	rye	0.4515	0.0203	47	296	159	0.5016	0.7717	1.0925	PE	M
Baking Motivation: Lower Cost	yes	spelt	1.4043	0.0358	4	51	159	0.2360	0.6005	0.5396	NE	M
Baking Motivation: Lower Cost	yes	wheat	0.2688	0.0157	91	602	159	0.6042	0.8333	1.0401	PE	M
Baking Motivation: Lower Cost	yes	wholeme al	3.4224	0.0550	56	319	162	0.0643	0.2931	1.2256	PE	M
Baking Motivation: Lower Cost	yes	organic: yes	0.2001	0.0134	96	680	162	0.6547	0.8510	0.9691	NE	M
Baking Motivation: Taste	yes	endosper m	0.7697	0.0261	499	638	896	0.3803	0.6722	0.9873	NE	M
Baking Motivation: Taste	yes	mix	2.7965	0.0497	123	145	896	0.0945	0.3264	1.0708	PE	M
Baking Motivation: Taste	yes	organic: no	0.7548	0.0261	342	432	896	0.3850	0.6416	0.9825	NE	M
Baking Motivation: Taste	yes	other	1.5951	0.0382	123	145	882	0.2066	0.5903	1.0522	PE	M
Baking Motivation: Taste	yes	other	33.4556	0.1720	10	29	896	0.0000	0.0000	0.4353	NE	M
Baking Motivation: Taste	yes	rye	1.0181	0.0305	245	296	882	0.3130	0.6260	1.0267	PE	M
Baking Motivation: Taste	yes	spelt	4.1534	0.0616	35	51	882	0.0416	0.1511	0.8512	NE	M
Baking Motivation: Taste	yes	wheat	0.8068	0.0272	479	602	882	0.3691	0.6418	0.9869	NE	M
Baking Motivation: Taste	yes	wholeme al	3.0837	0.0522	264	319	896	0.0791	0.3005	1.0446	PE	M
Baking Motivation: Taste	yes	organic: yes	0.7548	0.0261	554	680	896	0.3850	0.6416	1.0111	PE	M
Baking Motivation: Tradition	yes	endosper m	3.2968	0.0540	54	638	81	0.0694	0.2931	1.1818	PE	M
Baking Motivation: Tradition	yes	mix	0.9900	0.0296	7	145	81	0.3197	0.6231	0.6741	NE	M
Baking Motivation: Tradition	yes	organic: no	0.8823	0.0282	27	432	81	0.3476	0.6416	0.8580	NE	M
Baking Motivation: Tradition	yes	other	1.1296	0.0321	7	145	80	0.2879	0.6260	0.6602	NE	M
Baking Motivation: Tradition	yes	rye	13.1166	0.1095	36	296	80	0.0003	0.0093	1.6632	PE	M
Baking Motivation: Tradition	yes	spelt	4.3140	0.0628	8	51	80	0.0378	0.1511	2.1451	PE	M

Baking Motivation: Tradition	yes	wheat	11.4924	0.1025	29	602	80	0.0007	0.0093	0.6588	NE	M
Baking Motivation: Tradition	yes	wholemeal	0.3615	0.0179	20	319	81	0.5477	0.7633	0.8754	NE	M
Baking Motivation: Tradition	yes	organic: yes	0.8823	0.0282	54	680	81	0.3476	0.6416	1.0902	PE	M
Perceived Benefit: Better Digestibility	yes	endosperm	0.5907	0.0229	307	638	532	0.4421	0.6918	1.0230	PE	M
Perceived Benefit: Better Digestibility	yes	mix	0.1672	0.0122	71	145	532	0.6826	0.8437	1.0410	PE	M
Perceived Benefit: Better Digestibility	yes	organic: no	0.1530	0.0117	203	432	532	0.6956	0.8510	0.9822	NE	M
Perceived Benefit: Better Digestibility	yes	other	0.0135	0.0035	71	145	527	0.9075	0.9628	1.0165	PE	M
Perceived Benefit: Better Digestibility	yes	other	11.8702	0.1024	4	29	532	0.0006	0.0054	0.2932	NE	M
Perceived Benefit: Better Digestibility	yes	rye	0.0154	0.0038	144	296	527	0.9012	0.9628	1.0099	PE	M
Perceived Benefit: Better Digestibility	yes	spelt	0.0004	0.0006	24	51	527	0.9845	1.0000	0.9769	NE	M
Perceived Benefit: Better Digestibility	yes	wheat	0.0330	0.0055	288	602	527	0.8558	0.9628	0.9931	NE	M
Perceived Benefit: Better Digestibility	yes	organic: yes	0.1530	0.0117	329	680	532	0.6956	0.8510	1.0113	PE	M
Perceived Benefit: Gut Health	yes	endosperm	3.3650	0.0545	70	638	143	0.0666	0.2931	0.8678	NE	M
Perceived Benefit: Gut Health	yes	mix	12.4166	0.1048	32	145	143	0.0004	0.0054	1.7455	PE	M
Perceived Benefit: Gut Health	yes	organic: no	1.2381	0.0334	49	432	143	0.2658	0.6416	0.8820	NE	M
Perceived Benefit: Gut Health	yes	other	11.9370	0.1045	32	145	140	0.0006	0.0093	1.7245	PE	M
Perceived Benefit: Gut Health	yes	rye	1.6890	0.0393	31	296	140	0.1937	0.5903	0.8184	NE	M
Perceived Benefit: Gut Health	yes	spelt	0.7178	0.0256	9	51	140	0.3969	0.6615	1.3790	PE	M
Perceived Benefit: Gut Health	yes	wheat	2.4130	0.0470	68	602	140	0.1203	0.4011	0.8827	NE	M
Perceived Benefit: Gut Health	yes	wholemeal	0.0275	0.0049	39	319	143	0.8684	0.9072	0.9669	NE	M
Perceived Benefit: Gut Health	yes	organic: yes	1.2381	0.0334	94	680	143	0.2658	0.6416	1.0749	PE	M
Perceived Benefit: Healthier Ingredients	yes	endosperm	0.0215	0.0044	67	638	121	0.8833	0.9072	0.9816	NE	M
Perceived Benefit: Healthier Ingredients	yes	mix	0.3355	0.0172	13	145	121	0.5625	0.7633	0.8380	NE	M
Perceived Benefit: Healthier Ingredients	yes	organic: no	0.0887	0.0089	45	432	121	0.7659	0.8510	0.9573	NE	M
Perceived Benefit: Healthier Ingredients	yes	other	0.4709	0.0207	13	145	120	0.4926	0.7717	0.8174	NE	M

Perceived Benefit: Healthier Ingredients	yes	rye	5.7720	0.0726	44	296	120	0.0163	0.1234	1.3552	PE	M
Perceived Benefit: Healthier Ingredients	yes	spelt	0.9236	0.0291	3	51	120	0.3365	0.6410	0.5363	NE	M
Perceived Benefit: Healthier Ingredients	yes	wheat	1.1579	0.0325	60	602	120	0.2819	0.6260	0.9086	NE	M
Perceived Benefit: Healthier Ingredients	yes	wholemeal	1.3188	0.0341	40	319	121	0.2508	0.5776	1.1721	PE	M
Perceived Benefit: Healthier Ingredients	yes	organic: yes	0.0887	0.0089	76	680	121	0.7659	0.8510	1.0271	PE	M
Perceived Benefit: Intolerance/Allergy	yes	endosperm	1.1340	0.0317	49	638	78	0.2869	0.5893	1.1136	PE	M
Perceived Benefit: Intolerance/Allergy	yes	mix	1.5092	0.0365	6	145	78	0.2193	0.5554	0.6000	NE	M
Perceived Benefit: Intolerance/Allergy	yes	organic: no	2.2294	0.0448	37	432	78	0.1354	0.4514	1.2210	PE	M
Perceived Benefit: Intolerance/Allergy	yes	other	1.3795	0.0355	6	145	74	0.2402	0.6005	0.6117	NE	M
Perceived Benefit: Intolerance/Allergy	yes	other	0.1378	0.0110	3	29	78	0.7105	0.8437	1.5000	PE	M
Perceived Benefit: Intolerance/Allergy	yes	rye	5.2786	0.0695	29	296	74	0.0216	0.1234	1.4484	PE	M
Perceived Benefit: Intolerance/Allergy	yes	wheat	1.0431	0.0309	36	602	74	0.3071	0.6260	0.8841	NE	M
Perceived Benefit: Intolerance/Allergy	yes	wholemeal	0.1530	0.0116	20	319	78	0.6957	0.8437	0.9091	NE	M
Perceived Benefit: Intolerance/Allergy	yes	organic: yes	2.2294	0.0448	41	680	78	0.1354	0.4514	0.8596	NE	M
Perceived Benefit: Nutrient Availability	yes	endosperm	2.1426	0.0435	78	638	154	0.1433	0.4188	0.8979	NE	M
Perceived Benefit: Nutrient Availability	yes	mix	0.0385	0.0058	21	145	154	0.8445	0.9072	1.0636	PE	M
Perceived Benefit: Nutrient Availability	yes	organic: no	15.8165	0.1193	37	432	154	0.0001	0.0007	0.6184	NE	M
Perceived Benefit: Nutrient Availability	yes	other	0.0257	0.0048	21	145	150	0.8726	0.9628	1.0563	PE	M
Perceived Benefit: Nutrient Availability	yes	other	0.0606	0.0073	3	29	154	0.8056	0.9004	0.7597	NE	M
Perceived Benefit: Nutrient Availability	yes	rye	0.1436	0.0115	43	296	150	0.7048	0.9094	1.0595	PE	M
Perceived Benefit: Nutrient Availability	yes	spelt	0.3873	0.0188	5	51	150	0.5337	0.7907	0.7150	NE	M
Perceived Benefit: Nutrient Availability	yes	wheat	0.0338	0.0056	81	602	150	0.8540	0.9628	0.9813	NE	M
Perceived Benefit: Nutrient Availability	yes	wholemeal	2.4140	0.0462	52	319	154	0.1203	0.3808	1.1972	PE	M
Perceived Benefit: Nutrient Availability	yes	organic: yes	15.8165	0.1193	117	680	154	0.0001	0.0007	1.2424	PE	M

Supplementary Table 4. PERMANOVA results based on the Jaccard distance matrix of aroma profiles, with variables ranked by explained variance (R^2) (related to Figure 5D).

Variable	*Pseudo-F*	*R²*	*Sample Size*	*Number of Groups*	*Uncorrected p-value*	*Corrected p-value (BH-FDR)*
plants	2.5745	0.0046	557	2	0.0020	0.0116
flour_organic	2.0455	0.0037	553	2	0.0020	0.0116
flour_quality	1.9707	0.0035	557	4	0.0010	0.0079
flour_type_red_red	1.8579	0.0034	550	4	0.0010	0.0079
acid_beverage	1.8652	0.0033	557	2	0.0070	0.0338
lower_cost	1.8248	0.0033	557	2	0.0090	0.0412
samp_source	1.8068	0.0032	557	3	0.0010	0.0079
tradition	1.7394	0.0031	557	2	0.0200	0.0757
sustainanbility	1.7141	0.0031	557	2	0.0130	0.0566
sample_received_season	1.6674	0.0030	557	4	0.0020	0.0116
flour_change_origin	0.9861	0.0030	331	131	0.7450	0.8102
flour_type_red	1.6309	0.0029	557	9	0.0010	0.0079
stor_feeding	1.5949	0.0029	557	6	0.0010	0.0079
kids	1.5851	0.0028	555	2	0.0450	0.1554
europe_region	1.5390	0.0028	553	4	0.0030	0.0154
no_pets	1.5412	0.0028	557	2	0.0500	0.1554
cheese	1.5134	0.0027	557	2	0.0470	0.1554
dairy_products	1.4868	0.0027	557	2	0.0520	0.1560
bread_refresh_loc	1.1126	0.0027	417	44	0.0020	0.0116
bread_baked	1.4641	0.0026	556	2	0.0680	0.1849
cs_info	1.3232	0.0026	517	13	0.0030	0.0154
fermented_cereals	1.4224	0.0025	557	2	0.0640	0.1796
samp_month	1.1011	0.0025	432	12	0.1400	0.3085
flour_type	1.3964	0.0025	557	15	0.0010	0.0079

health_benefits_no_answer	1.3880	0.0025	557	2	0.0980	0.2508
alcoholic_beverage	1.3445	0.0024	557	2	0.1010	0.2511
dough_source	1.3317	0.0024	557	5	0.0200	0.0757
after_feeding	1.2735	0.0024	533	3	0.0940	0.2478
like_taste	1.3268	0.0024	557	2	0.1410	0.3085
dog	1.3182	0.0024	557	2	0.1560	0.3085
skill	1.2411	0.0023	528	5	0.0620	0.1796
hub	1.3026	0.0023	557	5	0.0230	0.0834
yeast_usage_prior	1.3009	0.0023	557	2	0.1550	0.3085
stable_glucose_level	1.2920	0.0023	557	2	0.1490	0.3085
samp_date	1.0825	0.0023	469	176	0.0010	0.0079
samp_country	1.2846	0.0023	557	19	0.0010	0.0079
beer	1.2845	0.0023	557	2	0.1530	0.3085
wholemeal_spelt	1.2839	0.0023	557	2	0.1290	0.3033
other_fermentations_yes_no	1.2171	0.0023	533	2	0.2160	0.3871
aroma_dough_difficulty	1.2398	0.0023	543	6	0.0200	0.0757
ph_measured	1.2268	0.0022	556	2	0.1900	0.3673
cow	1.2172	0.0022	557	2	0.1560	0.3085
lamas	1.2166	0.0022	557	2	0.0490	0.1554
fermented_vegetables	1.2118	0.0022	557	2	0.2020	0.3739
fermented_legumes	1.1959	0.0021	557	2	0.2320	0.4037
water_type	1.1823	0.0021	557	5	0.1260	0.3033
person_id	1.1782	0.0021	557	455	0.0010	0.0079
flour_change	1.1766	0.0021	557	2	0.2380	0.4060
hamster	1.1556	0.0021	557	2	0.2180	0.3871
intoleranaces_allergy	1.1519	0.0021	557	2	0.2430	0.4066
email_signup	1.0489	0.0020	521	2	0.3680	0.5426
fermented_roots	1.1122	0.0020	557	2	0.2010	0.3739

cat	1.0993	0.0020	557	2	0.3230	0.5006
samp_city	1.0983	0.0020	557	357	0.0010	0.0079
covid	1.0971	0.0020	557	3	0.2970	0.4785
health	1.0903	0.0020	557	2	0.3280	0.5006
guinea_pig	1.0857	0.0019	557	2	0.3230	0.5006
baking_no_answer	1.0756	0.0019	557	2	0.2550	0.4186
fish	1.0500	0.0019	557	2	0.3390	0.5085
rabbit	1.0252	0.0018	557	2	0.4040	0.5858
mental_health	1.0246	0.0018	557	2	0.4220	0.6019
dough_stor_device	1.0116	0.0018	557	3	0.4580	0.6325
dough_cover	1.0052	0.0018	557	5	0.4500	0.6315
goat	0.9861	0.0018	557	2	0.5560	0.7329
nutrient_availability	0.9858	0.0018	557	2	0.4960	0.6743
flour_additions	0.9709	0.0017	557	3	0.5650	0.7337
bird	0.9608	0.0017	557	2	0.6340	0.7454
condiment	0.9455	0.0017	557	2	0.5490	0.7329
wine	0.9178	0.0016	557	2	0.5820	0.7446
chicken	0.9157	0.0016	557	2	0.5910	0.7452
hobby	0.9079	0.0016	557	2	0.6090	0.7454
gebril	0.9022	0.0016	557	2	0.7880	0.8464
reptiles	0.8919	0.0016	557	2	0.6300	0.7454
other_fermentations	0.8904	0.0016	557	2	0.6170	0.7454
fermented_fruits	0.8883	0.0016	557	2	0.6620	0.7578
business_job	0.8703	0.0016	557	2	0.6520	0.7563
like_baking	0.8575	0.0015	557	2	0.6290	0.7454
bees	0.8415	0.0015	557	2	0.7320	0.8102
others_health_adv	0.8271	0.0015	557	2	0.7400	0.8102
gut_health	0.7900	0.0014	557	2	0.7320	0.8102

other_baking_reasons	0.7851	0.0014	557	2	0.7980	0.8467
more_satiety	0.7604	0.0014	557	2	0.8080	0.8469
horse	0.7432	0.0013	557	2	0.8910	0.9120
better_digestibility	0.7230	0.0013	557	2	0.8470	0.8773
healthier_ingredients	0.5923	0.0011	557	2	0.9520	0.9560
intolance_allergy	0.5907	0.0011	557	2	0.9560	0.9560

Supplementary Table 5. Mantel test results based on the Jaccard distance matrix of aroma profiles, with variables ranked by Pearson correlation coefficient (R^2) (related to Figure 5D). p-values were corrected using the Benjamini–Hochberg false discovery rate (BH-FDR).

Numerical Variable	***Number of samples***	***Mantel R (Pearson)***	***P-Value (Pearson)***	***Corrected P-Value (Pearson)***	***Mantel R (Spearman)***	***P-Value (Spearman)***	***Corrected P-Value (Spearman)***
number_sourdoughs	533	0.0666	0.0010	0.0165	0.0793	0.0010	0.0165
barley	557	0.0269	0.1050	0.3729	0.0229	0.2030	0.5913
rye	557	0.0211	0.0900	0.3729	0.0152	0.2950	0.6084
wheat	557	0.0201	0.0010	0.0165	0.0191	0.0010	0.0165
gluten_free_flour	557	0.0200	0.2260	0.5327	-0.0091	0.6390	0.8168
tta_laboratory	541	0.0193	0.1320	0.3960	0.0168	0.1150	0.3960
time_feeding	553	0.0180	0.2700	0.5940	0.0076	0.5810	0.8168
longitude	557	0.0165	0.2900	0.5981	0.0046	0.7340	0.8352
temp_feeding	557	0.0130	0.3540	0.6872	0.0220	0.0830	0.3913
ph_value_citizen	544	0.0089	0.4720	0.8653	0.0056	0.6350	0.8168
spelt	557	0.0087	0.5710	0.9422	-0.0032	0.8540	0.8960
bread_density	446	0.0073	0.7010	0.9529	0.0291	0.0430	0.3548
wholemeal_rye	557	0.0050	0.6650	0.9529	-0.0031	0.8170	0.8960
dough_age	556	0.0030	0.8370	0.9529	0.0117	0.3560	0.6782
refreshment_amount	557	0.0027	0.8860	0.9529	-0.0116	0.4110	0.6782
samp_year	556	0.0027	0.8630	0.9529	0.0097	0.4540	0.7134
refreshment_flour	557	0.0018	0.8980	0.9529	-0.0248	0.0750	0.3913

refreshment_water	557	0.0017	0.9170	0.9529	-0.0249	0.0690	0.3913
latitude	557	0.0012	0.9240	0.9529	0.0049	0.6870	0.8168
other_wheat_varieties	557	-0.0007	0.9760	0.9760	-0.0026	0.8960	0.8960
wholemeal_wheat	557	-0.0028	0.8370	0.9529	-0.0060	0.6930	0.8168
dough_yield	555	-0.0039	0.8030	0.9529	0.0022	0.8850	0.8960
elevation	557	-0.0041	0.6870	0.9529	-0.0049	0.5720	0.8168
temp_storage	557	-0.0070	0.6260	0.9529	-0.0055	0.6850	0.8168
pH_laboratory	544	-0.0177	0.1820	0.5005	-0.0145	0.2670	0.6084
bread_making_freque ncy	520	-0.0197	0.2050	0.5204	-0.0173	0.2410	0.6084
semi-wholemeal_wheat	557	-0.0241	0.1040	0.3729	-0.0269	0.1140	0.3960
sample_transport_dur	526	-0.0254	0.1130	0.3729	0.0147	0.2150	0.5913
bread_ph	444	-0.0297	0.0580	0.3190	-0.0124	0.3850	0.6782
dough_size_feeding	557	-0.0338	0.0550	0.3190	-0.0148	0.2770	0.6084
backslop_freq	554	-0.0447	0.0020	0.0220	-0.0398	0.0050	0.0550

Supplementary Table 6. Pearson and Spearman correlations, as well as cosine similarity, between perceived aromas and measurable sourdough characteristics, fermentation parameters, and substrate proportions per sourdough (related to Figure 5E–F). p-values were corrected using the Benjamini–Hochberg false discovery rate (BH-FDR).

Variable 1	***Variable 2***	***Pearson Correlation***	***Spearman Correlation***	***Cosine Similarity***	***Uncorrected Pearson P-Value***	***Corrected Pearson P-Value***	***Uncorrected Spearman P-Value***	***Corrected Spearman P-Value***
Chemical	bread_density	-0.0529	-0.0120	0.1779	0.2646	0.6083	0.8007	0.9132
Chemical	dough_age	-0.0334	-0.0200	0.1232	0.4316	0.7512	0.6374	0.8274
Chemical	dough_yield	0.0178	-0.0333	0.4013	0.6752	0.8637	0.4330	0.6924
Chemical	pH_laboratory	0.0027	-0.0073	0.4084	0.9507	0.9690	0.8648	0.9472
Chemical	backslop_freq	-0.0608	-0.0655	0.2290	0.1529	0.4941	0.1234	0.3561
Chemical	barley	-0.0191	0.0139	0.0021	0.6534	0.8573	0.7426	0.8870
Chemical	bread_ph	-0.0808	-0.1042	0.4005	0.0890	0.3569	0.0281	0.1240
Chemical	dough_size_feed ing	0.0416	0.0540	0.1356	0.3272	0.6586	0.2031	0.4653
Chemical	ph_value_citizen	-0.1017	-0.1070	0.3939	0.0177	0.1127	0.0125	0.0693

Chemical	rye	-0.0689	-0.0599	0.1263	0.1041	0.4000	0.1581	0.3963
Chemical	semi-wholemeal_wheat	0.0218	0.0236	0.1391	0.6075	0.8397	0.5785	0.7897
Chemical	spelt	0.0574	0.0849	0.1522	0.1759	0.5269	0.0452	0.1791
Chemical	temp_feeding	-0.0343	-0.0372	0.3886	0.4190	0.7353	0.3813	0.6475
Chemical	temp_storage	-0.0107	-0.0016	0.3227	0.8008	0.9062	0.9696	0.9900
Chemical	time_feeding	-0.0321	0.0108	0.2249	0.4514	0.7524	0.7996	0.9132
Chemical	wheat	0.1212	0.1237	0.3710	0.0042	0.0330	0.0035	0.0240
Chemical	wholemeal_rye	-0.0816	-0.0973	0.1269	0.0544	0.2477	0.0216	0.1065
Chemical	wholemeal_spelt	0.0217	0.0316	0.0767	0.6090	0.8397	0.4563	0.7081
Chemical	wholemeal_wheat	-0.0528	-0.0638	0.1163	0.2131	0.5581	0.1328	0.3719
Chemical	tta_laboratory	-0.1353	-0.1396	0.3404	0.0016	0.0144	0.0011	0.0089
Earthy	bread_density	-0.0188	0.0020	0.1936	0.6924	0.8657	0.9671	0.9890
Earthy	dough_age	-0.0314	-0.0467	0.1148	0.4597	0.7600	0.2720	0.5439
Earthy	dough_yield	0.0030	0.0262	0.3752	0.9443	0.9690	0.5372	0.7640
Earthy	pH_laboratory	0.0330	0.0430	0.3807	0.4420	0.7524	0.3170	0.5925
Earthy	backslop_freq	0.0159	-0.0191	0.2625	0.7094	0.8746	0.6529	0.8388
Earthy	barley	-0.0181	0.0235	0.0016	0.6704	0.8637	0.5805	0.7897
Earthy	bread_ph	-0.0578	-0.0600	0.3788	0.2243	0.5721	0.2067	0.4702
Earthy	dough_size_feeding	-0.0074	-0.0266	0.0857	0.8615	0.9294	0.5316	0.7594
Earthy	ph_value_citizen	-0.0055	0.0018	0.3775	0.8987	0.9523	0.9664	0.9890
Earthy	rye	-0.0825	-0.0383	0.1026	0.0518	0.2452	0.3667	0.6356
Earthy	semi-wholemeal_wheat	-0.0161	-0.0005	0.0980	0.7045	0.8720	0.9903	0.9976
Earthy	spelt	0.0449	0.0592	0.1351	0.2897	0.6360	0.1631	0.4062
Earthy	temp_feeding	-0.0297	-0.0118	0.3639	0.4844	0.7805	0.7804	0.9080
Earthy	temp_storage	-0.0139	-0.0608	0.2997	0.7439	0.8872	0.1519	0.3906
Earthy	time_feeding	0.0675	0.0310	0.2817	0.1127	0.4203	0.4670	0.7176
Earthy	wheat	0.0352	0.0321	0.2983	0.4071	0.7217	0.4496	0.7046
Earthy	wholemeal_rye	-0.0179	-0.0078	0.1655	0.6734	0.8637	0.8539	0.9422
Earthy	wholemeal_spelt	0.0447	0.0487	0.0943	0.2926	0.6400	0.2512	0.5382
Earthy	wholemeal_wheat	0.0439	0.0621	0.1876	0.3008	0.6404	0.1433	0.3794
Earthy	tta_laboratory	-0.0030	-0.0372	0.3554	0.9444	0.9690	0.3877	0.6498

Fermented	bread_density	-0.0975	-0.0533	0.3188	0.0396	0.2093	0.2612	0.5410
Fermented	dough_age	-0.0808	-0.0584	0.2115	0.0570	0.2529	0.1687	0.4116
Fermented	dough_yield	0.0054	-0.0387	0.6980	0.8994	0.9523	0.3624	0.6324
Fermented	pH_laboratory	0.0533	0.0489	0.7095	0.2148	0.5592	0.2553	0.5410
Fermented	backslop_freq	0.0531	0.0602	0.4982	0.2123	0.5581	0.1572	0.3963
Fermented	barley	-0.0467	-0.0574	0.0011	0.2707	0.6177	0.1762	0.4213
Fermented	bread_ph	0.0118	0.0298	0.7123	0.8045	0.9062	0.5308	0.7594
Fermented	dough_size_feeding	0.1002	0.0589	0.2407	0.0181	0.1138	0.1654	0.4102
Fermented	ph_value_citizen	0.0233	0.0237	0.7119	0.5872	0.8393	0.5817	0.7897
Fermented	rye	0.0574	0.0547	0.3545	0.1762	0.5269	0.1973	0.4536
Fermented	semi-wholemeal_wheat	0.1274	0.1188	0.2952	0.0026	0.0218	0.0050	0.0331
Fermented	spelt	0.0452	0.0626	0.2077	0.2873	0.6348	0.1401	0.3771
Fermented	temp_feeding	-0.1156	-0.1246	0.6716	0.0063	0.0461	0.0032	0.0225
Fermented	temp_storage	-0.0258	-0.0304	0.5621	0.5440	0.8239	0.4734	0.7221
Fermented	time_feeding	0.0367	0.1025	0.4514	0.3887	0.7021	0.0159	0.0823
Fermented	wheat	-0.0292	-0.0263	0.5002	0.4921	0.7909	0.5358	0.7637
Fermented	wholemeal_rye	-0.0645	-0.0455	0.2956	0.1283	0.4489	0.2840	0.5590
Fermented	wholemeal_spelt	0.0415	0.0460	0.1286	0.3278	0.6586	0.2784	0.5522
Fermented	wholemeal_wheat	-0.0321	-0.0402	0.2594	0.4496	0.7524	0.3431	0.6106
Fermented	tta_laboratory	-0.0846	-0.0863	0.6430	0.0492	0.2404	0.0448	0.1786
Fermented dairy	bread_density	-0.0747	-0.1565	0.3280	0.1152	0.4269	0.0009	0.0077
Fermented dairy	dough_age	0.0521	0.0609	0.2991	0.2197	0.5628	0.1515	0.3906
Fermented dairy	dough_yield	-0.0444	-0.0407	0.6940	0.2966	0.6404	0.3381	0.6087
Fermented dairy	pH_laboratory	0.0112	0.0029	0.7093	0.7948	0.9062	0.9463	0.9838
Fermented dairy	backslop_freq	0.1793	0.1542	0.5666	0.0000	0.0004	0.0003	0.0029
Fermented dairy	barley	-0.0462	-0.0248	0.0016	0.2760	0.6210	0.5590	0.7823
Fermented dairy	bread_ph	-0.0335	-0.0525	0.7023	0.4818	0.7795	0.2692	0.5439
Fermented dairy	dough_size_feeding	0.0973	0.0404	0.2390	0.0217	0.1301	0.3413	0.6095
Fermented dairy	ph_value_citizen	-0.0495	-0.0472	0.7044	0.2487	0.6002	0.2717	0.5439

Fermented dairy	rye	-0.0253	-0.0060	0.3034	0.5514	0.8251	0.8873	0.9602
Fermented dairy	semi-wholemeal_wheat	0.0217	0.0160	0.2245	0.6090	0.8397	0.7066	0.8577
Fermented dairy	spelt	-0.0465	-0.0351	0.1457	0.2735	0.6198	0.4077	0.6637
Fermented dairy	temp_feeding	-0.0377	-0.0357	0.6861	0.3744	0.6917	0.4008	0.6575
Fermented dairy	temp_storage	0.0508	0.0049	0.5955	0.2316	0.5828	0.9084	0.9730
Fermented dairy	time_feeding	0.0197	0.0020	0.4431	0.6442	0.8526	0.9630	0.9881
Fermented dairy	wheat	0.0834	0.0737	0.5563	0.0490	0.2404	0.0822	0.2655
Fermented dairy	wholemeal_rye	-0.0083	0.0231	0.3316	0.8444	0.9203	0.5858	0.7903
Fermented dairy	wholemeal_spelt	-0.0197	-0.0166	0.0861	0.6425	0.8526	0.6956	0.8554
Fermented dairy	wholemeal_wheat	0.0463	0.0773	0.3109	0.2751	0.6210	0.0683	0.2379
Fermented dairy	tta_laboratory	-0.0582	-0.0676	0.6512	0.1763	0.5269	0.1165	0.3473
Fruity	bread_density	-0.0683	-0.0529	0.2604	0.1501	0.4898	0.2649	0.5419
Fruity	dough_age	0.0026	-0.0688	0.2208	0.9511	0.9690	0.1049	0.3240
Fruity	dough_yield	-0.0437	-0.0025	0.5705	0.3046	0.6419	0.9525	0.9870
Fruity	pH_laboratory	0.0249	0.0215	0.5830	0.5627	0.8342	0.6175	0.8156
Fruity	backslop_freq	-0.0264	-0.0171	0.3746	0.5354	0.8188	0.6886	0.8554
Fruity	barley	0.0419	0.0755	0.0620	0.3239	0.6586	0.0749	0.2523
Fruity	bread_ph	-0.0157	-0.0330	0.5876	0.7421	0.8872	0.4877	0.7333
Fruity	dough_size_feeding	-0.0105	0.0741	0.1342	0.8050	0.9062	0.0806	0.2646
Fruity	ph_value_citizen	-0.0412	-0.0376	0.5836	0.3374	0.6658	0.3813	0.6475
Fruity	rye	-0.0230	-0.0163	0.2469	0.5875	0.8393	0.7005	0.8577
Fruity	semi-wholemeal_wheat	-0.0385	-0.0224	0.1435	0.3644	0.6783	0.5985	0.7972
Fruity	spelt	-0.0423	-0.0182	0.1132	0.3191	0.6586	0.6686	0.8479
Fruity	temp_feeding	0.0069	0.0290	0.5725	0.8706	0.9322	0.4943	0.7383
Fruity	temp_storage	-0.0216	-0.0567	0.4639	0.6116	0.8397	0.1818	0.4290
Fruity	time_feeding	0.0874	-0.0049	0.4135	0.0399	0.2093	0.9078	0.9730
Fruity	wheat	0.0601	0.0560	0.4593	0.1564	0.5004	0.1867	0.4358

Fruity	wholemeal_rye	0.0050	-0.0113	0.2815	0.9055	0.9540	0.7908	0.9132
Fruity	wholemeal_spelt	-0.0583	-0.0583	0.0357	0.1697	0.5241	0.1692	0.4116
Fruity	wholemeal_wheat	-0.0549	-0.0325	0.1911	0.1957	0.5316	0.4446	0.7002
Fruity	tta_laboratory	-0.0068	-0.0410	0.5436	0.8748	0.9325	0.3415	0.6095
Grain/Cereal	bread_density	-0.0262	-0.0731	0.3869	0.5816	0.8366	0.1233	0.3561
Grain/Cereal	dough_age	0.0265	0.0742	0.2972	0.5335	0.8188	0.0806	0.2646
Grain/Cereal	dough_yield	0.0558	0.1133	0.7505	0.1895	0.5311	0.0075	0.0448
Grain/Cereal	pH_laboratory	0.0722	0.0844	0.7532	0.0924	0.3685	0.0491	0.1876
Grain/Cereal	backslop_freq	0.0331	0.0960	0.5169	0.4364	0.7512	0.0239	0.1131
Grain/Cereal	barley	-0.0487	-0.0233	0.0042	0.2510	0.6014	0.5837	0.7903
Grain/Cereal	bread_ph	0.0302	-0.0086	0.7552	0.5253	0.8151	0.8561	0.9429
Grain/Cereal	dough_size_feeding	-0.0172	-0.0272	0.1721	0.6850	0.8657	0.5214	0.7569
Grain/Cereal	ph_value_citizen	0.0791	0.0629	0.7591	0.0651	0.2808	0.1427	0.3794
Grain/Cereal	rye	-0.1300	-0.1027	0.2624	0.0021	0.0185	0.0153	0.0805
Grain/Cereal	semi-wholemeal_wheat	-0.0413	-0.0419	0.1968	0.3305	0.6586	0.3231	0.5969
Grain/Cereal	spelt	-0.0341	0.0124	0.1664	0.4214	0.7374	0.7698	0.9032
Grain/Cereal	temp_feeding	-0.0384	-0.1168	0.7285	0.3661	0.6783	0.0058	0.0367
Grain/Cereal	temp_storage	-0.0179	-0.0297	0.6025	0.6735	0.8637	0.4836	0.7333
Grain/Cereal	time_feeding	-0.0603	-0.0172	0.4271	0.1565	0.5004	0.6870	0.8554
Grain/Cereal	wheat	-0.0277	-0.0438	0.5348	0.5149	0.8057	0.3018	0.5761
Grain/Cereal	wholemeal_rye	0.0620	0.0847	0.3931	0.1442	0.4802	0.0457	0.1799
Grain/Cereal	wholemeal_spelt	-0.0569	-0.0560	0.0689	0.1800	0.5269	0.1868	0.4358
Grain/Cereal	wholemeal_wheat	0.0853	0.0871	0.3496	0.0442	0.2227	0.0398	0.1654
Grain/Cereal	tta_laboratory	-0.0195	0.0233	0.7003	0.6517	0.8571	0.5886	0.7906
Nutty	bread_density	-0.0564	-0.0525	0.0988	0.2343	0.5843	0.2686	0.5439

Nutty	dough_age	-0.0304	-0.0161	0.0728	0.4738	0.7736	0.7056	0.8577
Nutty	dough_yield	0.0376	0.0827	0.2673	0.3769	0.6926	0.0515	0.1943
Nutty	pH_laboratory	-0.0588	-0.0404	0.2647	0.1710	0.5249	0.3470	0.6123
Nutty	backslop_freq	0.0123	0.0233	0.1874	0.7721	0.8958	0.5849	0.7903
Nutty	barley	-0.0093	0.0585	0.0039	0.8268	0.9186	0.1676	0.4116
Nutty	bread_ph	-0.0479	-0.0904	0.2684	0.3136	0.6519	0.0569	0.2073
Nutty	dough_size_feeding	-0.0170	0.0252	0.0493	0.6896	0.8657	0.5527	0.7772
Nutty	ph_value_citizen	0.0242	0.0201	0.2722	0.5732	0.8365	0.6393	0.8274
Nutty	rye	-0.0481	-0.0245	0.0791	0.2573	0.6021	0.5636	0.7823
Nutty	semi-wholemeal_wheat	0.0351	0.0296	0.1115	0.4081	0.7217	0.4860	0.7333
Nutty	spelt	-0.0486	0.0138	0.0216	0.2523	0.6021	0.7448	0.8870
Nutty	temp_feeding	0.0263	0.0072	0.2668	0.5350	0.8188	0.8657	0.9472
Nutty	temp_storage	0.0536	0.0485	0.2471	0.2062	0.5482	0.2531	0.5397
Nutty	time_feeding	-0.0199	0.0315	0.1485	0.6399	0.8526	0.4604	0.7126
Nutty	wheat	0.1030	0.0972	0.2629	0.0150	0.0973	0.0218	0.1065
Nutty	wholemeal_rye	-0.0325	-0.0052	0.0995	0.4441	0.7524	0.9034	0.9712
Nutty	wholemeal_spelt	0.0105	0.0105	0.0477	0.8047	0.9062	0.8047	0.9132
Nutty	wholemeal_wheat	-0.0050	-0.0044	0.1015	0.9054	0.9540	0.9172	0.9730
Nutty	tta_laboratory	-0.0087	-0.0031	0.2484	0.8400	0.9203	0.9429	0.9837
Sour	bread_density	0.0446	0.1060	0.3371	0.3478	0.6661	0.0252	0.1150
Sour	dough_age	-0.0228	0.0024	0.1941	0.5912	0.8397	0.9557	0.9871
Sour	dough_yield	-0.0137	0.0149	0.5556	0.7467	0.8872	0.7259	0.8711
Sour	pH_laboratory	-0.0045	0.0041	0.5697	0.9170	0.9578	0.9241	0.9752
Sour	backslop_freq	0.0086	-0.0238	0.3809	0.8399	0.9203	0.5768	0.7897
Sour	barley	0.0474	0.1039	0.0662	0.2640	0.6083	0.0141	0.0759
Sour	bread_ph	-0.0464	-0.0377	0.5729	0.3290	0.6586	0.4277	0.6857
Sour	dough_size_feeding	0.0451	0.0584	0.1738	0.2882	0.6348	0.1686	0.4116
Sour	ph_value_citizen	-0.0896	-0.0805	0.5611	0.0367	0.2012	0.0606	0.2181
Sour	rye	0.0114	0.0063	0.2632	0.7882	0.9062	0.8827	0.9588
Sour	semi-wholemeal_wheat	-0.0579	-0.0471	0.1220	0.1725	0.5249	0.2668	0.5439
Sour	spelt	0.0404	0.0861	0.1738	0.3411	0.6661	0.0423	0.1707

Sour	temp_feeding	0.0399	-0.0075	0.5597	0.3475	0.6661	0.8594	0.9449
Sour	temp_storage	-0.0251	0.0114	0.4463	0.5540	0.8261	0.7879	0.9125
Sour	time_feeding	0.0046	0.0415	0.3494	0.9148	0.9578	0.3301	0.6010
Sour	wheat	-0.1338	-0.1487	0.3359	0.0015	0.0143	0.0004	0.0044
Sour	wholemeal_rye	0.1294	0.1386	0.3626	0.0022	0.0190	0.0010	0.0084
Sour	wholemeal_spelt	0.0319	0.0312	0.1057	0.4523	0.7524	0.4625	0.7141
Sour	wholemeal_whe at	-0.0523	-0.0668	0.1846	0.2175	0.5628	0.1153	0.3458
Sour	tta_laboratory	0.1207	0.1331	0.5690	0.0049	0.0380	0.0019	0.0146
Toasted	bread_density	-0.0471	-0.0500	0.1293	0.3209	0.6586	0.2918	0.5639
Toasted	dough_age	-0.0213	0.0290	0.1016	0.6162	0.8397	0.4957	0.7383
Toasted	dough_yield	0.0485	0.1271	0.3260	0.2541	0.6021	0.0027	0.0194
Toasted	pH_laboratory	-0.0422	-0.0152	0.3211	0.3262	0.6586	0.7236	0.8711
Toasted	backslop_freq	0.0769	-0.0156	0.2691	0.0705	0.3002	0.7133	0.8625
Toasted	barley	0.1067	0.0381	0.1164	0.0117	0.0802	0.3692	0.6356
Toasted	bread_ph	-0.0199	-0.0310	0.3177	0.6757	0.8637	0.5148	0.7508
Toasted	dough_size_feed ing	-0.0272	0.0305	0.0534	0.5218	0.8117	0.4728	0.7221
Toasted	ph_value_citizen	-0.0444	-0.0538	0.3208	0.3009	0.6404	0.2100	0.4758
Toasted	rye	-0.0149	0.0155	0.1323	0.7248	0.8867	0.7149	0.8628
Toasted	semi-wholemeal_whe at	0.0827	0.0838	0.1701	0.0511	0.2437	0.0482	0.1852
Toasted	spelt	-0.0176	-0.0005	0.0643	0.6784	0.8637	0.9915	0.9976
Toasted	temp_feeding	0.0284	0.0462	0.3201	0.5035	0.7969	0.2765	0.5512
Toasted	temp_storage	0.0484	-0.0046	0.2876	0.2544	0.6021	0.9139	0.9730
Toasted	time_feeding	0.0462	0.0613	0.2317	0.2779	0.6215	0.1501	0.3891
Toasted	wheat	-0.0058	0.0037	0.2303	0.8912	0.9472	0.9309	0.9791
Toasted	wholemeal_rye	-0.0374	-0.0170	0.1216	0.3783	0.6928	0.6888	0.8554
Toasted	wholemeal_spelt	0.0646	0.0368	0.1059	0.1277	0.4489	0.3863	0.6498
Toasted	wholemeal_whe at	-0.0434	-0.0367	0.0898	0.3070	0.6426	0.3878	0.6498
Toasted	tta_laboratory	0.0303	-0.0342	0.3136	0.4824	0.7795	0.4270	0.6857
Veggie	bread_density	-0.0280	0.0744	0.2107	0.5547	0.8261	0.1169	0.3473
Veggie	dough_age	0.0219	-0.0287	0.1849	0.6057	0.8397	0.4988	0.7397
Veggie	dough_yield	-0.0112	0.0300	0.4386	0.7916	0.9062	0.4803	0.7308
Veggie	pH_laboratory	0.0605	0.0739	0.4447	0.1591	0.5036	0.0850	0.2718

Veggie	backslop_freq	-0.0803	-0.0921	0.2425	0.0588	0.2592	0.0303	0.1324
Veggie	barley	-0.0240	-0.0455	0.0000	0.5718	0.8365	0.2837	0.5590
Veggie	bread_ph	-0.0162	-0.0320	0.4308	0.7328	0.8872	0.5008	0.7397
Veggie	dough_size_feeding	0.0230	0.1038	0.1285	0.5873	0.8393	0.0142	0.0759
Veggie	ph_value_citizen	0.0028	-0.0042	0.4463	0.9482	0.9690	0.9227	0.9752
Veggie	rye	-0.0084	-0.0094	0.1940	0.8435	0.9203	0.8252	0.9218
Veggie	semi-wholemeal_wheat	-0.0196	-0.0137	0.1152	0.6439	0.8526	0.7477	0.8888
Veggie	spelt	-0.0147	0.0024	0.0988	0.7291	0.8872	0.9546	0.9871
Veggie	temp_feeding	-0.0560	-0.0331	0.4238	0.1866	0.5271	0.4352	0.6925
Veggie	temp_storage	-0.0778	-0.0451	0.3202	0.0667	0.2858	0.2881	0.5634
Veggie	time_feeding	-0.0362	-0.0039	0.2452	0.3953	0.7096	0.9279	0.9775
Veggie	wheat	-0.0217	-0.0072	0.3110	0.6089	0.8397	0.8660	0.9472
Veggie	wholemeal_rye	0.0664	0.0480	0.2640	0.1176	0.4313	0.2579	0.5410
Veggie	wholemeal_spelt	0.0236	0.0189	0.0836	0.5790	0.8366	0.6564	0.8388
Veggie	wholemeal_wheat	-0.0321	-0.0219	0.1502	0.4492	0.7524	0.6068	0.8031
Veggie	tta_laboratory	0.0038	0.0135	0.4167	0.9291	0.9643	0.7532	0.8920

Supplementary Table 7. Pearson and Spearman correlations, as well as cosine similarity, between measurable sourdough characteristics, home experiment results, fermentation parameters, and substrate proportions per sourdough (related to Figure 6E). p-values were corrected using the Benjamini–Hochberg false discovery rate (BH-FDR).

Variable 1	***Variable 2***	***Pearson Correlation***	***Spearman Correlation***	***Cosine Similarity***	***Uncorrected Pearson P-Value***	***Corrected Pearson P-Value***	***Uncorrected Spearman P-Value***	***Corrected Spearman P-Value***
bread_density	dough_age	-0.0170	0.0071	0.1880	0.7213	0.8844	0.8815	0.9588
bread_density	dough_yield	0.0089	0.1312	0.5179	0.8524	0.9243	0.0056	0.0364
bread_density	pH_laboratory	0.0485	0.0432	0.5254	0.3126	0.6519	0.3692	0.6356
bread_density	backslop_freq	-0.0691	-0.2610	0.3081	0.1463	0.4802	0.0000	0.0000
bread_density	barley	-0.0182	-0.0730	0.0180	0.7020	0.8720	0.1238	0.3561
bread_density	bread_ph	-0.0023	0.0266	0.5177	0.9614	0.9706	0.5787	0.7897
bread_density	dough_size_feeding	-0.0194	-0.0203	0.1040	0.6833	0.8657	0.6689	0.8479
bread_density	ph_value_citizen	-0.0049	-0.0888	0.5202	0.9181	0.9578	0.0621	0.2212

bread_density	rye	0.0284	0.1110	0.2604	0.5495	0.8251	0.0190	0.0964
bread_density	semi-wholemeal_wheat	-0.0659	-0.1419	0.0883	0.1646	0.5159	0.0027	0.0194
bread_density	spelt	0.0349	0.1040	0.1612	0.4626	0.7609	0.0281	0.1240
bread_density	temp_feeding	0.0117	-0.0266	0.5155	0.8054	0.9062	0.5754	0.7897
bread_density	temp_storage	0.0654	0.0941	0.4589	0.1679	0.5237	0.0470	0.1828
bread_density	time_feeding	-0.0336	0.0861	0.2895	0.4804	0.7795	0.0703	0.2425
bread_density	wheat	-0.0694	-0.3064	0.3443	0.1435	0.4802	0.0000	0.0000
bread_density	wholemeal_rye	0.1042	0.2153	0.3289	0.0279	0.1567	0.0000	0.0001
bread_density	wholemeal_spelt	-0.0154	0.0653	0.0616	0.7453	0.8872	0.1684	0.4116
bread_density	wholemeal_wheat	-0.0156	-0.0067	0.1915	0.7427	0.8872	0.8881	0.9602
bread_density	tta_laboratory	0.0166	0.2313	0.5007	0.7305	0.8872	0.0000	0.0000
dough_age	bread_density	-0.0170	0.0071	0.1880	0.7213	0.8844	0.8815	0.9588
dough_age	dough_yield	0.0184	0.0476	0.3702	0.6651	0.8633	0.2636	0.5410
dough_age	pH_laboratory	0.0439	-0.0795	0.4767	0.3068	0.6426	0.0642	0.2257
dough_age	backslop_freq	0.0759	0.0383	0.2983	0.0745	0.3116	0.3688	0.6356
dough_age	barley	-0.0188	-0.0867	0.0005	0.6583	0.8587	0.0410	0.1668
dough_age	bread_ph	0.0097	-0.0192	0.3755	0.8389	0.9203	0.6875	0.8554
dough_age	dough_size_feeding	0.0364	-0.0128	0.1234	0.3915	0.7047	0.7631	0.8992
dough_age	ph_value_citizen	-0.0419	0.0137	0.3621	0.3294	0.6586	0.7503	0.8902
dough_age	rye	-0.0164	0.0045	0.1541	0.6989	0.8720	0.9160	0.9730
dough_age	semi-wholemeal_wheat	0.0136	-0.0630	0.1223	0.7492	0.8872	0.1377	0.3771
dough_age	spelt	-0.0544	-0.1531	0.0443	0.2001	0.5386	0.0003	0.0031
dough_age	temp_feeding	0.0198	0.0162	0.3674	0.6418	0.8526	0.7023	0.8577
dough_age	temp_storage	0.0701	0.0368	0.3398	0.0989	0.3869	0.3871	0.6498
dough_age	time_feeding	-0.0002	0.0030	0.2267	0.9965	0.9965	0.9447	0.9837
dough_age	wheat	0.0022	-0.0106	0.2720	0.9579	0.9706	0.8032	0.9132

dough_age	wholemeal_rye	0.0214	0.0943	0.1944	0.6151	0.8397	0.0262	0.1188
dough_age	wholemeal_spelt	-0.0132	-0.0128	0.0403	0.7564	0.8883	0.7632	0.8992
dough_age	wholemeal_whe at	0.0835	0.0247	0.2188	0.0492	0.2404	0.5613	0.7823
dough_age	tta_laboratory	0.0448	0.1080	0.4586	0.2984	0.6404	0.0120	0.0671
dough_yield	bread_density	0.0089	0.1312	0.5179	0.8524	0.9243	0.0056	0.0364
dough_yield	dough_age	0.0184	0.0476	0.3702	0.6651	0.8633	0.2636	0.5410
dough_yield	pH_laboratory	0.0395	0.0192	0.9833	0.3582	0.6739	0.6550	0.8388
dough_yield	backslop_freq	-0.0498	-0.0745	0.6483	0.2432	0.5916	0.0804	0.2646
dough_yield	barley	0.0372	0.0615	0.0529	0.3817	0.6970	0.1481	0.3872
dough_yield	bread_ph	-0.0983	-0.1069	0.9729	0.0388	0.2069	0.0246	0.1141
dough_yield	dough_size_fee ding	-0.0100	-0.0656	0.2370	0.8146	0.9099	0.1225	0.3561
dough_yield	ph_value_citizen	-0.0323	-0.1101	0.9793	0.4526	0.7524	0.0103	0.0591
dough_yield	rye	0.1453	0.1883	0.4659	0.0006	0.0064	0.0000	0.0001
dough_yield	semi-wholemeal_whe at	-0.0567	-0.0444	0.2820	0.1823	0.5269	0.2965	0.5695
dough_yield	spelt	-0.0879	-0.0912	0.2313	0.0384	0.2069	0.0316	0.1356
dough_yield	temp_feeding	-0.1016	-0.0629	0.9551	0.0167	0.1070	0.1389	0.3771
dough_yield	temp_storage	-0.0133	-0.0767	0.7949	0.7549	0.8883	0.0708	0.2425
dough_yield	time_feeding	0.0960	0.2029	0.6113	0.0242	0.1427	0.0000	0.0000
dough_yield	wheat	-0.1986	-0.2476	0.6911	0.0000	0.0001	0.0000	0.0000
dough_yield	wholemeal_rye	0.0713	0.1441	0.4767	0.0933	0.3697	0.0007	0.0065
dough_yield	wholemeal_spelt	-0.0504	-0.0813	0.1298	0.2354	0.5843	0.0556	0.2038
dough_yield	wholemeal_whe at	0.0332	0.0741	0.3955	0.4357	0.7512	0.0813	0.2650
dough_yield	tta_laboratory	0.0564	0.1431	0.9246	0.1910	0.5311	0.0009	0.0075
elevation	bread_density	0.0220	0.1449	0.4049	0.6433	0.8526	0.0022	0.0159
elevation	dough_age	-0.0351	-0.0792	0.2629	0.4089	0.7217	0.0621	0.2212
elevation	dough_yield	-0.1040	-0.1969	0.7382	0.0143	0.0947	0.0000	0.0001
elevation	pH_laboratory	-0.1163	-0.1412	0.7610	0.0066	0.0480	0.0010	0.0079

elevation	backslop_freq	-0.0207	-0.0183	0.4969	0.6274	0.8501	0.6678	0.8479
elevation	barley	-0.0514	-0.1171	0.0033	0.2261	0.5721	0.0057	0.0364
elevation	bread_ph	0.1097	0.1146	0.7510	0.0207	0.1255	0.0157	0.0815
elevation	dough_size_feeding	0.0812	0.1148	0.2360	0.0554	0.2477	0.0067	0.0412
elevation	ph_value_citizen	0.0576	0.1121	0.7627	0.1801	0.5269	0.0089	0.0518
elevation	rye	0.1045	0.0932	0.4026	0.0136	0.0917	0.0279	0.1240
elevation	semi-wholemeal_wheat	0.1618	0.1840	0.3252	0.0001	0.0016	0.0000	0.0002
elevation	spelt	0.2026	0.1436	0.3173	0.0000	0.0000	0.0007	0.0065
elevation	temp_feeding	-0.0134	0.0430	0.7396	0.7521	0.8878	0.3109	0.5865
elevation	temp_storage	-0.0078	0.0663	0.6125	0.8542	0.9247	0.1183	0.3489
elevation	time_feeding	-0.0762	-0.0600	0.4229	0.0734	0.3105	0.1585	0.3963
elevation	wheat	-0.2388	-0.2317	0.4463	0.0000	0.0000	0.0000	0.0000
elevation	wholemeal_rye	0.0397	0.0488	0.3835	0.3500	0.6662	0.2499	0.5373
elevation	wholemeal_spelt	0.0242	0.0201	0.1225	0.5690	0.8365	0.6352	0.8274
elevation	wholemeal_wheat	0.0176	0.0330	0.3116	0.6787	0.8637	0.4364	0.6925
elevation	tta_laboratory	0.0138	0.0721	0.7215	0.7491	0.8872	0.0938	0.2953
pH_laboratory	bread_density	0.0485	0.0432	0.5254	0.3126	0.6519	0.3692	0.6356
pH_laboratory	dough_age	0.0439	-0.0795	0.4767	0.3068	0.6426	0.0642	0.2257
pH_laboratory	dough_yield	0.0395	0.0192	0.9833	0.3582	0.6739	0.6550	0.8388
pH_laboratory	backslop_freq	-0.0826	-0.0999	0.6594	0.0550	0.2477	0.0201	0.1013
pH_laboratory	barley	-0.0076	-0.0264	0.0482	0.8593	0.9285	0.5388	0.7645
pH_laboratory	bread_ph	-0.0712	-0.0565	0.9897	0.1395	0.4802	0.2413	0.5279
pH_laboratory	dough_size_feeding	-0.0266	0.0501	0.2405	0.5351	0.8188	0.2433	0.5284
pH_laboratory	ph_value_citizen	0.0694	0.0182	0.9948	0.1102	0.4179	0.6764	0.8520
pH_laboratory	rye	-0.0200	-0.0530	0.4474	0.6418	0.8526	0.2168	0.4878
pH_laboratory	semi-wholemeal_wheat	-0.0954	-0.0853	0.2912	0.0261	0.1510	0.0467	0.1827

pH_laboratory	spelt	0.0577	0.0104	0.2551	0.1789	0.5269	0.8089	0.9132
pH_laboratory	temp_feeding	-0.0457	-0.0814	0.9722	0.2875	0.6348	0.0579	0.2095
pH_laboratory	temp_storage	-0.0762	-0.1100	0.8063	0.0759	0.3148	0.0102	0.0591
pH_laboratory	time_feeding	-0.0039	-0.0967	0.6058	0.9285	0.9643	0.0246	0.1141
pH_laboratory	wheat	-0.0554	-0.0691	0.7215	0.1970	0.5328	0.1073	0.3298
pH_laboratory	wholemeal_rye	0.0574	0.0762	0.4797	0.1813	0.5269	0.0756	0.2535
pH_laboratory	wholemeal_spelt	0.0509	0.0793	0.1445	0.2356	0.5843	0.0645	0.2257
pH_laboratory	wholemeal_whe at	-0.0416	-0.0423	0.3906	0.3324	0.6586	0.3251	0.5989
pH_laboratory	tta_laboratory	-0.1745	-0.1448	0.9321	0.0000	0.0007	0.0007	0.0067
backslop_freq	bread_density	-0.0691	-0.2610	0.3081	0.1463	0.4802	0.0000	0.0000
backslop_freq	dough_age	0.0759	0.0383	0.2983	0.0745	0.3116	0.3688	0.6356
backslop_freq	dough_yield	-0.0498	-0.0745	0.6483	0.2432	0.5916	0.0804	0.2646
backslop_freq	pH_laboratory	-0.0826	-0.0999	0.6594	0.0550	0.2477	0.0201	0.1013
backslop_freq	barley	-0.0289	-0.0572	0.0104	0.4971	0.7969	0.1791	0.4258
backslop_freq	bread_ph	-0.0318	0.0003	0.6664	0.5047	0.7969	0.9955	0.9976
backslop_freq	dough_size_fee ding	0.2458	0.0971	0.3383	0.0000	0.0000	0.0222	0.1068
backslop_freq	ph_value_citizen	0.0418	0.0635	0.6633	0.3324	0.6586	0.1400	0.3771
backslop_freq	rye	-0.1377	-0.1170	0.2032	0.0012	0.0112	0.0058	0.0367
backslop_freq	semi- wholemeal_whe at	0.0414	0.0758	0.2264	0.3308	0.6586	0.0747	0.2523
backslop_freq	spelt	-0.0895	-0.0657	0.1015	0.0351	0.1940	0.1225	0.3561
backslop_freq	temp_feeding	0.0152	-0.0166	0.6497	0.7216	0.8844	0.6966	0.8554
backslop_freq	temp_storage	0.5154	0.1991	0.7639	0.0000	0.0000	0.0000	0.0000
backslop_freq	time_feeding	-0.0402	-0.0501	0.3796	0.3472	0.6661	0.2407	0.5279
backslop_freq	wheat	0.1424	0.0957	0.5566	0.0008	0.0081	0.0243	0.1141
backslop_freq	wholemeal_rye	-0.0941	-0.0634	0.2525	0.0268	0.1519	0.1358	0.3769
backslop_freq	wholemeal_spelt	0.0095	0.0298	0.1007	0.8230	0.9161	0.4845	0.7333
backslop_freq	wholemeal_whe at	0.0495	0.0745	0.2975	0.2448	0.5931	0.0796	0.2646

backslop_freq	tta_laboratory	-0.0955	-0.0701	0.5955	0.0267	0.1519	0.1046	0.3240
barley	bread_density	-0.0182	-0.0730	0.0180	0.7020	0.8720	0.1238	0.3561
barley	dough_age	-0.0188	-0.0867	0.0005	0.6583	0.8587	0.0410	0.1668
barley	dough_yield	0.0372	0.0615	0.0529	0.3817	0.6970	0.1481	0.3872
barley	pH_laboratory	-0.0076	-0.0264	0.0482	0.8593	0.9285	0.5388	0.7645
barley	backslop_freq	-0.0289	-0.0572	0.0104	0.4971	0.7969	0.1791	0.4258
barley	bread_ph	-0.0758	-0.0534	0.0534	0.1109	0.4179	0.2617	0.5410
barley	dough_size_fee ding	-0.0068	0.0227	0.0051	0.8730	0.9322	0.5932	0.7929
barley	ph_value_citizen	-0.1507	-0.0961	0.0342	0.0004	0.0047	0.0250	0.1149
barley	rye	-0.0239	0.0278	0.0003	0.5742	0.8365	0.5121	0.7492
barley	semi-wholemeal_whe at	-0.0034	0.0420	0.0109	0.9354	0.9690	0.3228	0.5969
barley	spelt	-0.0084	0.1197	0.0039	0.8431	0.9203	0.0047	0.0317
barley	temp_feeding	0.0650	0.0426	0.0614	0.1252	0.4489	0.3158	0.5922
barley	temp_storage	-0.0221	-0.0248	0.0259	0.6026	0.8397	0.5588	0.7823
barley	time_feeding	0.1939	0.0492	0.1830	0.0000	0.0001	0.2478	0.5347
barley	wheat	-0.0477	-0.0205	0.0022	0.2614	0.6076	0.6287	0.8235
barley	wholemeal_rye	-0.0259	-0.0478	0.0000	0.5419	0.8229	0.2599	0.5410
barley	wholemeal_spelt	-0.0068	-0.0121	0.0000	0.8723	0.9322	0.7762	0.9073
barley	wholemeal_whe at	-0.0207	-0.0398	0.0000	0.6256	0.8494	0.3483	0.6129
barley	tta_laboratory	0.2350	0.0278	0.1281	0.0000	0.0000	0.5189	0.7550
bread_ph	bread_density	-0.0023	0.0266	0.5177	0.9614	0.9706	0.5787	0.7897
bread_ph	dough_age	0.0097	-0.0192	0.3755	0.8389	0.9203	0.6875	0.8554
bread_ph	dough_yield	-0.0983	-0.1069	0.9729	0.0388	0.2069	0.0246	0.1141
bread_ph	pH_laboratory	-0.0712	-0.0565	0.9897	0.1395	0.4802	0.2413	0.5279
bread_ph	backslop_freq	-0.0318	0.0003	0.6664	0.5047	0.7969	0.9955	0.9976
bread_ph	barley	-0.0758	-0.0534	0.0534	0.1109	0.4179	0.2617	0.5410
bread_ph	dough_size_fee ding	-0.0211	0.0489	0.2134	0.6582	0.8587	0.3042	0.5789

bread_ph	ph_value_citizen	0.2152	0.2655	0.9898	0.0000	0.0001	0.0000	0.0000
bread_ph	rye	-0.0119	-0.0268	0.4460	0.8025	0.9062	0.5730	0.7897
bread_ph	semi-wholemeal_wheat	-0.0106	0.0132	0.2679	0.8231	0.9161	0.7816	0.9080
bread_ph	spelt	-0.0560	-0.0053	0.2396	0.2392	0.5879	0.9106	0.9730
bread_ph	temp_feeding	-0.0523	-0.1461	0.9685	0.2716	0.6177	0.0020	0.0152
bread_ph	temp_storage	-0.0584	-0.0284	0.8023	0.2196	0.5628	0.5512	0.7771
bread_ph	time_feeding	-0.0239	-0.0409	0.6411	0.6171	0.8397	0.3921	0.6500
bread_ph	wheat	-0.0887	-0.1046	0.7152	0.0617	0.2701	0.0276	0.1240
bread_ph	wholemeal_rye	0.0248	0.0755	0.4779	0.6018	0.8397	0.1121	0.3394
bread_ph	wholemeal_spelt	0.0294	0.0102	0.1450	0.5362	0.8188	0.8306	0.9256
bread_ph	wholemeal_wheat	0.0226	0.0136	0.3911	0.6348	0.8526	0.7751	0.9073
bread_ph	tta_laboratory	-0.0060	0.0015	0.9350	0.9012	0.9526	0.9745	0.9934
dough_size_feeding	bread_density	-0.0194	-0.0203	0.1040	0.6833	0.8657	0.6689	0.8479
dough_size_feeding	dough_age	0.0364	-0.0128	0.1234	0.3915	0.7047	0.7631	0.8992
dough_size_feeding	dough_yield	-0.0100	-0.0656	0.2370	0.8146	0.9099	0.1225	0.3561
dough_size_feeding	pH_laboratory	-0.0266	0.0501	0.2405	0.5351	0.8188	0.2433	0.5284
dough_size_feeding	backslop_freq	0.2458	0.0971	0.3383	0.0000	0.0000	0.0222	0.1068
dough_size_feeding	barley	-0.0068	0.0227	0.0051	0.8730	0.9322	0.5932	0.7929
dough_size_feeding	bread_ph	-0.0211	0.0489	0.2134	0.6582	0.8587	0.3042	0.5789
dough_size_feeding	ph_value_citizen	-0.0179	0.0721	0.2364	0.6764	0.8637	0.0931	0.2947
dough_size_feeding	rye	-0.0322	-0.0642	0.0813	0.4482	0.7524	0.1304	0.3689
dough_size_feeding	semi-wholemeal_wheat	-0.0071	0.0103	0.0653	0.8675	0.9322	0.8087	0.9132
dough_size_feeding	spelt	0.0415	-0.0180	0.0997	0.3278	0.6586	0.6723	0.8491
dough_size_feeding	temp_feeding	0.0275	-0.0342	0.2428	0.5167	0.8057	0.4200	0.6802

dough_size_feeding	temp_storage	0.1935	0.0494	0.3069	0.0000	0.0001	0.2446	0.5295
dough_size_feeding	time_feeding	-0.0354	-0.1166	0.1206	0.4060	0.7217	0.0061	0.0378
dough_size_feeding	wheat	0.0489	0.0006	0.2091	0.2497	0.6005	0.9879	0.9976
dough_size_feeding	wholemeal_rye	-0.0201	-0.0417	0.0980	0.6356	0.8526	0.3263	0.5994
dough_size_feeding	wholemeal_spelt	0.0015	-0.0118	0.0356	0.9714	0.9790	0.7815	0.9080
dough_size_feeding	wholemeal_wheat	-0.0366	0.0548	0.0635	0.3888	0.7021	0.1963	0.4531
dough_size_feeding	tta_laboratory	-0.0233	-0.0348	0.2185	0.5894	0.8397	0.4188	0.6800
ph_value_citizen	bread_density	-0.0049	-0.0888	0.5202	0.9181	0.9578	0.0621	0.2212
ph_value_citizen	dough_age	-0.0419	0.0137	0.3621	0.3294	0.6586	0.7503	0.8902
ph_value_citizen	dough_yield	-0.0323	-0.1101	0.9793	0.4526	0.7524	0.0103	0.0591
ph_value_citizen	pH_laboratory	0.0694	0.0182	0.9948	0.1102	0.4179	0.6764	0.8520
ph_value_citizen	backslop_freq	0.0418	0.0635	0.6633	0.3324	0.6586	0.1400	0.3771
ph_value_citizen	barley	-0.1507	-0.0961	0.0342	0.0004	0.0047	0.0250	0.1149
ph_value_citizen	bread_ph	0.2152	0.2655	0.9898	0.0000	0.0001	0.0000	0.0000
ph_value_citizen	dough_size_feeding	-0.0179	0.0721	0.2364	0.6764	0.8637	0.0931	0.2947
ph_value_citizen	rye	-0.1535	-0.1693	0.4322	0.0003	0.0038	0.0001	0.0009
ph_value_citizen	semi-wholemeal_wheat	0.0164	0.0281	0.2992	0.7036	0.8720	0.5126	0.7492
ph_value_citizen	spelt	-0.0406	-0.0256	0.2449	0.3449	0.6661	0.5514	0.7771
ph_value_citizen	temp_feeding	0.0279	-0.0026	0.9714	0.5157	0.8057	0.9523	0.9870
ph_value_citizen	temp_storage	-0.0044	0.0069	0.8042	0.9182	0.9578	0.8732	0.9534
ph_value_citizen	time_feeding	-0.2002	-0.2445	0.6385	0.0000	0.0001	0.0000	0.0000
ph_value_citizen	wheat	0.1057	0.1155	0.7260	0.0137	0.0917	0.0070	0.0424
ph_value_citizen	wholemeal_rye	-0.0241	-0.0375	0.4722	0.5749	0.8365	0.3829	0.6485
ph_value_citizen	wholemeal_spelt	0.0192	0.0314	0.1434	0.6546	0.8573	0.4646	0.7157
ph_value_citizen	wholemeal_wheat	0.0245	0.0105	0.4006	0.5693	0.8365	0.8076	0.9132
ph_value_citizen	tta_laboratory	-0.3153	-0.2419	0.9221	0.0000	0.0000	0.0000	0.0000

rye	bread_density	0.0284	0.1110	0.2604	0.5495	0.8251	0.0190	0.0964
rye	dough_age	-0.0164	0.0045	0.1541	0.6989	0.8720	0.9160	0.9730
rye	dough_yield	0.1453	0.1883	0.4659	0.0006	0.0064	0.0000	0.0001
rye	pH_laboratory	-0.0200	-0.0530	0.4474	0.6418	0.8526	0.2168	0.4878
rye	backslop_freq	-0.1377	-0.1170	0.2032	0.0012	0.0112	0.0058	0.0367
rye	barley	-0.0239	0.0278	0.0003	0.5742	0.8365	0.5121	0.7492
rye	bread_ph	-0.0119	-0.0268	0.4460	0.8025	0.9062	0.5730	0.7897
rye	dough_size_fee ding	-0.0322	-0.0642	0.0813	0.4482	0.7524	0.1304	0.3689
rye	ph_value_citizen	-0.1535	-0.1693	0.4322	0.0003	0.0038	0.0001	0.0009
rye	semi-wholemeal_whe at	-0.0482	-0.0285	0.0917	0.2558	0.6021	0.5014	0.7397
rye	spelt	-0.0616	0.0199	0.0589	0.1463	0.4802	0.6387	0.8274
rye	temp_feeding	-0.0741	-0.0475	0.4230	0.0807	0.3323	0.2634	0.5410
rye	temp_storage	-0.0226	0.0629	0.3516	0.5944	0.8397	0.1384	0.3771
rye	time_feeding	0.1077	0.1831	0.3478	0.0113	0.0780	0.0000	0.0002
rye	wheat	-0.3320	-0.2938	0.1226	0.0000	0.0000	0.0000	0.0000
rye	wholemeal_rye	-0.0813	-0.1055	0.1491	0.0552	0.2477	0.0127	0.0695
rye	wholemeal_spelt	-0.0598	-0.0551	0.0102	0.1584	0.5036	0.1944	0.4520
rye	wholemeal_whe at	-0.1673	-0.1136	0.0405	0.0001	0.0010	0.0073	0.0438
rye	tta_laboratory	0.1384	0.1556	0.4629	0.0012	0.0119	0.0003	0.0030
semi-wholemeal_whe at	bread_density	-0.0659	-0.1419	0.0883	0.1646	0.5159	0.0027	0.0194
semi-wholemeal_whe at	dough_age	0.0136	-0.0630	0.1223	0.7492	0.8872	0.1377	0.3771
semi-wholemeal_whe at	dough_yield	-0.0567	-0.0444	0.2820	0.1823	0.5269	0.2965	0.5695
semi-wholemeal_whe at	pH_laboratory	-0.0954	-0.0853	0.2912	0.0261	0.1510	0.0467	0.1827

semi-wholemeal_wheat	backslop_freq	0.0414	0.0758	0.2264	0.3308	0.6586	0.0747	0.2523
semi-wholemeal_wheat	barley	-0.0034	0.0420	0.0109	0.9354	0.9690	0.3228	0.5969
semi-wholemeal_wheat	bread_ph	-0.0106	0.0132	0.2679	0.8231	0.9161	0.7816	0.9080
semi-wholemeal_wheat	dough_size_feeding	-0.0071	0.0103	0.0653	0.8675	0.9322	0.8087	0.9132
semi-wholemeal_wheat	ph_value_citizen	0.0164	0.0281	0.2992	0.7036	0.8720	0.5126	0.7492
semi-wholemeal_wheat	rye	-0.0482	-0.0285	0.0917	0.2558	0.6021	0.5014	0.7397
semi-wholemeal_wheat	spelt	-0.0280	0.0289	0.0479	0.5092	0.8020	0.4954	0.7383
semi-wholemeal_wheat	temp_feeding	0.0542	0.0576	0.2996	0.2014	0.5399	0.1744	0.4193
semi-wholemeal_wheat	temp_storage	0.0254	0.0622	0.2533	0.5495	0.8251	0.1424	0.3794
semi-wholemeal_wheat	time_feeding	-0.0254	-0.0161	0.1595	0.5504	0.8251	0.7049	0.8577
semi-wholemeal_wheat	wheat	-0.2035	-0.2100	0.0810	0.0000	0.0000	0.0000	0.0000
semi-wholemeal_wheat	wholemeal_rye	-0.0629	-0.0412	0.0872	0.1384	0.4789	0.3312	0.6010
semi-wholemeal_wheat	wholemeal_spelt	-0.0439	-0.0460	0.0000	0.3009	0.6404	0.2787	0.5522
semi-wholemeal_wheat	wholemeal_wheat	0.1408	0.1730	0.2404	0.0009	0.0089	0.0000	0.0006
semi-wholemeal_wheat	tta_laboratory	0.0322	0.0643	0.2887	0.4554	0.7550	0.1352	0.3769
spelt	bread_density	0.0349	0.1040	0.1612	0.4626	0.7609	0.0281	0.1240
spelt	dough_age	-0.0544	-0.1531	0.0443	0.2001	0.5386	0.0003	0.0031

spelt	dough_yield	-0.0879	-0.0912	0.2313	0.0384	0.2069	0.0316	0.1356
spelt	pH_laboratory	0.0577	0.0104	0.2551	0.1789	0.5269	0.8089	0.9132
spelt	backslop_freq	-0.0895	-0.0657	0.1015	0.0351	0.1940	0.1225	0.3561
spelt	barley	-0.0084	0.1197	0.0039	0.8431	0.9203	0.0047	0.0317
spelt	bread_ph	-0.0560	-0.0053	0.2396	0.2392	0.5879	0.9106	0.9730
spelt	dough_size_feeding	0.0415	-0.0180	0.0997	0.3278	0.6586	0.6723	0.8491
spelt	ph_value_citizen	-0.0406	-0.0256	0.2449	0.3449	0.6661	0.5514	0.7771
spelt	rye	-0.0616	0.0199	0.0589	0.1463	0.4802	0.6387	0.8274
spelt	semi-wholemeal_wheat	-0.0280	0.0289	0.0479	0.5092	0.8020	0.4954	0.7383
spelt	temp_feeding	-0.0360	-0.0449	0.2354	0.3968	0.7101	0.2898	0.5634
spelt	temp_storage	-0.0675	0.0412	0.1635	0.1114	0.4179	0.3320	0.6010
spelt	time_feeding	0.0557	0.0106	0.1953	0.1912	0.5311	0.8042	0.9132
spelt	wheat	-0.2059	-0.1767	0.0443	0.0000	0.0000	0.0000	0.0004
spelt	wholemeal_rye	-0.0982	-0.0966	0.0346	0.0204	0.1255	0.0226	0.1079
spelt	wholemeal_spelt	0.0843	0.0561	0.1159	0.0467	0.2337	0.1859	0.4358
spelt	wholemeal_wheat	-0.0550	-0.0281	0.0498	0.1947	0.5311	0.5080	0.7461
spelt	tta_laboratory	0.0289	0.0370	0.2470	0.5019	0.7969	0.3903	0.6500
temp_feeding	bread_density	0.0117	-0.0266	0.5155	0.8054	0.9062	0.5754	0.7897
temp_feeding	dough_age	0.0198	0.0162	0.3674	0.6418	0.8526	0.7023	0.8577
temp_feeding	dough_yield	-0.1016	-0.0629	0.9551	0.0167	0.1070	0.1389	0.3771
temp_feeding	pH_laboratory	-0.0457	-0.0814	0.9722	0.2875	0.6348	0.0579	0.2095
temp_feeding	backslop_freq	0.0152	-0.0166	0.6497	0.7216	0.8844	0.6966	0.8554
temp_feeding	barley	0.0650	0.0426	0.0614	0.1252	0.4489	0.3158	0.5922
temp_feeding	bread_ph	-0.0523	-0.1461	0.9685	0.2716	0.6177	0.0020	0.0152
temp_feeding	dough_size_feeding	0.0275	-0.0342	0.2428	0.5167	0.8057	0.4200	0.6802
temp_feeding	ph_value_citizen	0.0279	-0.0026	0.9714	0.5157	0.8057	0.9523	0.9870
temp_feeding	rye	-0.0741	-0.0475	0.4230	0.0807	0.3323	0.2634	0.5410

temp_feeding	semi-wholemeal_wheat	0.0542	0.0576	0.2996	0.2014	0.5399	0.1744	0.4193
temp_feeding	spelt	-0.0360	-0.0449	0.2354	0.3968	0.7101	0.2898	0.5634
temp_feeding	temp_storage	0.0590	0.0221	0.7957	0.1644	0.5159	0.6030	0.8003
temp_feeding	time_feeding	-0.3444	-0.3289	0.5340	0.0000	0.0000	0.0000	0.0000
temp_feeding	wheat	0.0552	0.0410	0.7161	0.1932	0.5311	0.3342	0.6032
temp_feeding	wholemeal_rye	0.0453	0.0290	0.4708	0.2863	0.6348	0.4942	0.7383
temp_feeding	wholemeal_spelt	-0.0499	-0.0005	0.1258	0.2398	0.5879	0.9909	0.9976
temp_feeding	wholemeal_wheat	0.0022	-0.0402	0.3859	0.9588	0.9706	0.3441	0.6107
temp_feeding	tta_laboratory	0.1179	0.1050	0.9211	0.0060	0.0448	0.0146	0.0772
temp_storage	bread_density	0.0654	0.0941	0.4589	0.1679	0.5237	0.0470	0.1828
temp_storage	dough_age	0.0701	0.0368	0.3398	0.0989	0.3869	0.3871	0.6498
temp_storage	dough_yield	-0.0133	-0.0767	0.7949	0.7549	0.8883	0.0708	0.2425
temp_storage	pH_laboratory	-0.0762	-0.1100	0.8063	0.0759	0.3148	0.0102	0.0591
temp_storage	backslop_freq	0.5154	0.1991	0.7639	0.0000	0.0000	0.0000	0.0000
temp_storage	barley	-0.0221	-0.0248	0.0259	0.6026	0.8397	0.5588	0.7823
temp_storage	bread_ph	-0.0584	-0.0284	0.8023	0.2196	0.5628	0.5512	0.7771
temp_storage	dough_size_feeding	0.1935	0.0494	0.3069	0.0000	0.0001	0.2446	0.5295
temp_storage	ph_value_citizen	-0.0044	0.0069	0.8042	0.9182	0.9578	0.8732	0.9534
temp_storage	rye	-0.0226	0.0629	0.3516	0.5944	0.8397	0.1384	0.3771
temp_storage	semi-wholemeal_wheat	0.0254	0.0622	0.2533	0.5495	0.8251	0.1424	0.3794
temp_storage	spelt	-0.0675	0.0412	0.1635	0.1114	0.4179	0.3320	0.6010
temp_storage	temp_feeding	0.0590	0.0221	0.7957	0.1644	0.5159	0.6030	0.8003
temp_storage	time_feeding	-0.0031	-0.0021	0.4905	0.9428	0.9690	0.9604	0.9881
temp_storage	wheat	0.0829	-0.0379	0.6209	0.0505	0.2428	0.3714	0.6376
temp_storage	wholemeal_rye	-0.0400	0.0706	0.3627	0.3462	0.6661	0.0959	0.3007
temp_storage	wholemeal_spelt	-0.0651	-0.0872	0.0758	0.1251	0.4489	0.0396	0.1654

temp_storage	wholemeal_wheat	-0.0313	-0.1047	0.3030	0.4608	0.7600	0.0134	0.0728
temp_storage	tta_laboratory	-0.0223	0.0749	0.7530	0.6046	0.8397	0.0816	0.2650
time_feeding	bread_density	-0.0336	0.0861	0.2895	0.4804	0.7795	0.0703	0.2425
time_feeding	dough_age	-0.0002	0.0030	0.2267	0.9965	0.9965	0.9447	0.9837
time_feeding	dough_yield	0.0960	0.2029	0.6113	0.0242	0.1427	0.0000	0.0000
time_feeding	pH_laboratory	-0.0039	-0.0967	0.6058	0.9285	0.9643	0.0246	0.1141
time_feeding	backslop_freq	-0.0402	-0.0501	0.3796	0.3472	0.6661	0.2407	0.5279
time_feeding	barley	0.1939	0.0492	0.1830	0.0000	0.0001	0.2478	0.5347
time_feeding	bread_ph	-0.0239	-0.0409	0.6411	0.6171	0.8397	0.3921	0.6500
time_feeding	dough_size_feeding	-0.0354	-0.1166	0.1206	0.4060	0.7217	0.0061	0.0378
time_feeding	ph_value_citizen	-0.2002	-0.2445	0.6385	0.0000	0.0001	0.0000	0.0000
time_feeding	rye	0.1077	0.1831	0.3478	0.0113	0.0780	0.0000	0.0002
time_feeding	semi-wholemeal_wheat	-0.0254	-0.0161	0.1595	0.5504	0.8251	0.7049	0.8577
time_feeding	spelt	0.0557	0.0106	0.1953	0.1912	0.5311	0.8042	0.9132
time_feeding	temp_feeding	-0.3444	-0.3289	0.5340	0.0000	0.0000	0.0000	0.0000
time_feeding	temp_storage	-0.0031	-0.0021	0.4905	0.9428	0.9690	0.9604	0.9881
time_feeding	wheat	-0.0863	-0.1671	0.3961	0.0426	0.2164	0.0001	0.0010
time_feeding	wholemeal_rye	0.0107	0.0606	0.2960	0.8020	0.9062	0.1546	0.3943
time_feeding	wholemeal_spelt	0.0408	0.0362	0.1179	0.3382	0.6658	0.3958	0.6527
time_feeding	wholemeal_wheat	-0.0193	0.0516	0.2277	0.6501	0.8568	0.2262	0.5035
time_feeding	tta_laboratory	0.2309	0.1451	0.6317	0.0000	0.0000	0.0007	0.0067
wheat	bread_density	-0.0694	-0.3064	0.3443	0.1435	0.4802	0.0000	0.0000
wheat	dough_age	0.0022	-0.0106	0.2720	0.9579	0.9706	0.8032	0.9132
wheat	dough_yield	-0.1986	-0.2476	0.6911	0.0000	0.0001	0.0000	0.0000
wheat	pH_laboratory	-0.0554	-0.0691	0.7215	0.1970	0.5328	0.1073	0.3298
wheat	backslop_freq	0.1424	0.0957	0.5566	0.0008	0.0081	0.0243	0.1141
wheat	barley	-0.0477	-0.0205	0.0022	0.2614	0.6076	0.6287	0.8235

wheat	bread_ph	-0.0887	-0.1046	0.7152	0.0617	0.2701	0.0276	0.1240
wheat	dough_size_fee ding	0.0489	0.0006	0.2091	0.2497	0.6005	0.9879	0.9976
wheat	ph_value_citizen	0.1057	0.1155	0.7260	0.0137	0.0917	0.0070	0.0424
wheat	rye	-0.3320	-0.2938	0.1226	0.0000	0.0000	0.0000	0.0000
wheat	semi- wholemeal_whe at	-0.2035	-0.2100	0.0810	0.0000	0.0000	0.0000	0.0000
wheat	spelt	-0.2059	-0.1767	0.0443	0.0000	0.0000	0.0000	0.0004
wheat	temp_feeding	0.0552	0.0410	0.7161	0.1932	0.5311	0.3342	0.6032
wheat	temp_storage	0.0829	-0.0379	0.6209	0.0505	0.2428	0.3714	0.6376
wheat	time_feeding	-0.0863	-0.1671	0.3961	0.0426	0.2164	0.0001	0.0010
wheat	wholemeal_rye	-0.4041	-0.3969	0.0997	0.0000	0.0000	0.0000	0.0000
wheat	wholemeal_spelt	-0.1184	-0.1121	0.0215	0.0051	0.0391	0.0081	0.0476
wheat	wholemeal_whe at	-0.2407	-0.2216	0.1353	0.0000	0.0000	0.0000	0.0000
wheat	tta_laboratory	-0.5197	-0.5854	0.5514	0.0000	0.0000	0.0000	0.0000
wholemeal_rye	bread_density	0.1042	0.2153	0.3289	0.0279	0.1567	0.0000	0.0001
wholemeal_rye	dough_age	0.0214	0.0943	0.1944	0.6151	0.8397	0.0262	0.1188
wholemeal_rye	dough_yield	0.0713	0.1441	0.4767	0.0933	0.3697	0.0007	0.0065
wholemeal_rye	pH_laboratory	0.0574	0.0762	0.4797	0.1813	0.5269	0.0756	0.2535
wholemeal_rye	backslop_freq	-0.0941	-0.0634	0.2525	0.0268	0.1519	0.1358	0.3769
wholemeal_rye	barley	-0.0259	-0.0478	0.0000	0.5419	0.8229	0.2599	0.5410
wholemeal_rye	bread_ph	0.0248	0.0755	0.4779	0.6018	0.8397	0.1121	0.3394
wholemeal_rye	dough_size_fee ding	-0.0201	-0.0417	0.0980	0.6356	0.8526	0.3263	0.5994
wholemeal_rye	ph_value_citizen	-0.0241	-0.0375	0.4722	0.5749	0.8365	0.3829	0.6485
wholemeal_rye	rye	-0.0813	-0.1055	0.1491	0.0552	0.2477	0.0127	0.0695
wholemeal_rye	semi- wholemeal_whe at	-0.0629	-0.0412	0.0872	0.1384	0.4789	0.3312	0.6010
wholemeal_rye	spelt	-0.0982	-0.0966	0.0346	0.0204	0.1255	0.0226	0.1079
wholemeal_rye	temp_feeding	0.0453	0.0290	0.4708	0.2863	0.6348	0.4942	0.7383

wholemeal_rye	temp_storage	-0.0400	0.0706	0.3627	0.3462	0.6661	0.0959	0.3007
wholemeal_rye	time_feeding	0.0107	0.0606	0.2960	0.8020	0.9062	0.1546	0.3943
wholemeal_rye	wheat	-0.4041	-0.3969	0.0997	0.0000	0.0000	0.0000	0.0000
wholemeal_rye	wholemeal_spelt	-0.0119	-0.0173	0.0562	0.7785	0.9016	0.6845	0.8554
wholemeal_rye	wholemeal_whe at	-0.0637	-0.0568	0.1360	0.1330	0.4628	0.1803	0.4271
wholemeal_rye	tta_laboratory	0.3352	0.3856	0.5503	0.0000	0.0000	0.0000	0.0000
wholemeal_spelt	bread_density	-0.0154	0.0653	0.0616	0.7453	0.8872	0.1684	0.4116
wholemeal_spelt	dough_age	-0.0132	-0.0128	0.0403	0.7564	0.8883	0.7632	0.8992
wholemeal_spelt	dough_yield	-0.0504	-0.0813	0.1298	0.2354	0.5843	0.0556	0.2038
wholemeal_spelt	pH_laboratory	0.0509	0.0793	0.1445	0.2356	0.5843	0.0645	0.2257
wholemeal_spelt	backslop_freq	0.0095	0.0298	0.1007	0.8230	0.9161	0.4845	0.7333
wholemeal_spelt	barley	-0.0068	-0.0121	0.0000	0.8723	0.9322	0.7762	0.9073
wholemeal_spelt	bread_ph	0.0294	0.0102	0.1450	0.5362	0.8188	0.8306	0.9256
wholemeal_spelt	dough_size_fee ding	0.0015	-0.0118	0.0356	0.9714	0.9790	0.7815	0.9080
wholemeal_spelt	ph_value_citizen	0.0192	0.0314	0.1434	0.6546	0.8573	0.4646	0.7157
wholemeal_spelt	rye	-0.0598	-0.0551	0.0102	0.1584	0.5036	0.1944	0.4520
wholemeal_spelt	semi-wholemeal_whe at	-0.0439	-0.0460	0.0000	0.3009	0.6404	0.2787	0.5522
wholemeal_spelt	spelt	0.0843	0.0561	0.1159	0.0467	0.2337	0.1859	0.4358
wholemeal_spelt	temp_feeding	-0.0499	-0.0005	0.1258	0.2398	0.5879	0.9909	0.9976
wholemeal_spelt	temp_storage	-0.0651	-0.0872	0.0758	0.1251	0.4489	0.0396	0.1654
wholemeal_spelt	time_feeding	0.0408	0.0362	0.1179	0.3382	0.6658	0.3958	0.6527
wholemeal_spelt	wheat	-0.1184	-0.1121	0.0215	0.0051	0.0391	0.0081	0.0476
wholemeal_spelt	wholemeal_rye	-0.0119	-0.0173	0.0562	0.7785	0.9016	0.6845	0.8554
wholemeal_spelt	wholemeal_whe at	-0.0009	-0.0002	0.0548	0.9837	0.9868	0.9966	0.9976
wholemeal_spelt	tta_laboratory	0.1281	0.1395	0.1781	0.0028	0.0232	0.0011	0.0089
wholemeal_whe at	bread_density	-0.0156	-0.0067	0.1915	0.7427	0.8872	0.8881	0.9602

wholemeal_wheat	dough_age	0.0835	0.0247	0.2188	0.0492	0.2404	0.5613	0.7823
wholemeal_wheat	dough_yield	0.0332	0.0741	0.3955	0.4357	0.7512	0.0813	0.2650
wholemeal_wheat	pH_laboratory	-0.0416	-0.0423	0.3906	0.3324	0.6586	0.3251	0.5989
wholemeal_wheat	backslop_freq	0.0495	0.0745	0.2975	0.2448	0.5931	0.0796	0.2646
wholemeal_wheat	barley	-0.0207	-0.0398	0.0000	0.6256	0.8494	0.3483	0.6129
wholemeal_wheat	bread_ph	0.0226	0.0136	0.3911	0.6348	0.8526	0.7751	0.9073
wholemeal_wheat	dough_size_feeding	-0.0366	0.0548	0.0635	0.3888	0.7021	0.1963	0.4531
wholemeal_wheat	ph_value_citizen	0.0245	0.0105	0.4006	0.5693	0.8365	0.8076	0.9132
wholemeal_wheat	rye	-0.1673	-0.1136	0.0405	0.0001	0.0010	0.0073	0.0438
wholemeal_wheat	semi-wholemeal_wheat	0.1408	0.1730	0.2404	0.0009	0.0089	0.0000	0.0006
wholemeal_wheat	spelt	-0.0550	-0.0281	0.0498	0.1947	0.5311	0.5080	0.7461
wholemeal_wheat	temp_feeding	0.0022	-0.0402	0.3859	0.9588	0.9706	0.3441	0.6107
wholemeal_wheat	temp_storage	-0.0313	-0.1047	0.3030	0.4608	0.7600	0.0134	0.0728
wholemeal_wheat	time_feeding	-0.0193	0.0516	0.2277	0.6501	0.8568	0.2262	0.5035
wholemeal_wheat	wheat	-0.2407	-0.2216	0.1353	0.0000	0.0000	0.0000	0.0000
wholemeal_wheat	wholemeal_rye	-0.0637	-0.0568	0.1360	0.1330	0.4628	0.1803	0.4271
wholemeal_wheat	wholemeal_spelt	-0.0009	-0.0002	0.0548	0.9837	0.9868	0.9966	0.9976
wholemeal_wheat	tta_laboratory	0.1727	0.1917	0.4247	0.0001	0.0008	0.0000	0.0001
tta_laboratory	bread_density	0.0166	0.2313	0.5007	0.7305	0.8872	0.0000	0.0000
tta_laboratory	dough_age	0.0448	0.1080	0.4586	0.2984	0.6404	0.0120	0.0671
tta_laboratory	dough_yield	0.0564	0.1431	0.9246	0.1910	0.5311	0.0009	0.0075
tta_laboratory	pH_laboratory	-0.1745	-0.1448	0.9321	0.0000	0.0007	0.0007	0.0067
tta_laboratory	backslop_freq	-0.0955	-0.0701	0.5955	0.0267	0.1519	0.1046	0.3240
tta_laboratory	barley	0.2350	0.0278	0.1281	0.0000	0.0000	0.5189	0.7550

tta_laboratory	bread_ph	-0.0060	0.0015	0.9350	0.9012	0.9526	0.9745	0.9934
tta_laboratory	dough_size_feeding	-0.0233	-0.0348	0.2185	0.5894	0.8397	0.4188	0.6800
tta_laboratory	ph_value_citizen	-0.3153	-0.2419	0.9221	0.0000	0.0000	0.0000	0.0000
tta_laboratory	rye	0.1384	0.1556	0.4629	0.0012	0.0119	0.0003	0.0030
tta_laboratory	semi-wholemeal_wheat	0.0322	0.0643	0.2887	0.4554	0.7550	0.1352	0.3769
tta_laboratory	spelt	0.0289	0.0370	0.2470	0.5019	0.7969	0.3903	0.6500
tta_laboratory	temp_feeding	0.1179	0.1050	0.9211	0.0060	0.0448	0.0146	0.0772
tta_laboratory	temp_storage	-0.0223	0.0749	0.7530	0.6046	0.8397	0.0816	0.2650
tta_laboratory	time_feeding	0.2309	0.1451	0.6317	0.0000	0.0000	0.0007	0.0067
tta_laboratory	wheat	-0.5197	-0.5854	0.5514	0.0000	0.0000	0.0000	0.0000
tta_laboratory	wholemeal_rye	0.3352	0.3856	0.5503	0.0000	0.0000	0.0000	0.0000
tta_laboratory	wholemeal_spelt	0.1281	0.1395	0.1781	0.0028	0.0232	0.0011	0.0089
tta_laboratory	wholemeal_wheat	0.1727	0.1917	0.4247	0.0001	0.0008	0.0000	0.0001

Supplementary Table 8. Chi-square enrichment test results for sourdough ingredients across countries (related to Figure 7A). *p*-values were corrected using the Benjamini–Hochberg false discovery rate (BH-FDR). Indicator codes: PE = positive enrichment, NE = negative enrichment, M = multiple indicators, U = unique indicator.

Feature	***Category***	***Cluster***	***Chi2 Statistic***	***Cramér's V***	***Cluster Count with Category***	***Cluster Total***	***Category Total***	***Raw p-value***	***Corrected p-value***	***Enrichment Score***	***Interpretation***	***Indicator***
Flour Additions	yes	Italy	21.5315	0.1398	16	81	74	0.0000	0.0000	2.9416	PE	M
Flour Change	yes	France	7.8217	0.0842	18	23	521	0.0052	0.0213	1.6553	PE	M
Flour Change	yes	Germany	17.3575	0.1255	50	158	521	0.0000	0.0002	0.6694	NE	M
Flour Milling Grade	endosperm	Finland	50.9589	0.2136	120	142	630	0.0000	0.0000	1.4983	PE	M
Flour Milling Grade	endosperm	Switzerland	18.6251	0.1291	187	393	630	0.0000	0.0001	0.8436	NE	M
Flour Milling Grade	endosperm	Italy	11.8811	0.1031	61	81	630	0.0006	0.0031	1.3352	PE	M
Flour Milling Grade	endosperm	Belgium	10.5508	0.0972	47	113	630	0.0012	0.0055	0.7374	NE	M
Flour Milling Grade	endosperm	Denmark	6.2889	0.0750	4	17	630	0.0121	0.0428	0.4172	NE	M

Flour Milling Grade	mix	Germany	7.2910	0.0808	9	158	141	0.0069	0.0252	0.4513	NE	M
Flour Milling Grade	mix	Denmark	21.8652	0.1399	9	17	141	0.0000	0.0000	4.1940	PE	M
Flour Milling Grade	mix	Sweden	10.5949	0.0974	9	25	141	0.0011	0.0055	2.8519	PE	M
Flour Milling Grade	mix	United Kingdom	4.8977	0.0662	5	14	141	0.0269	0.0905	2.8293	PE	M
Flour Milling Grade	other	Belgium	43.4672	0.1973	14	113	29	0.0000	0.0000	4.7720	PE	M
Flour Milling Grade	wholemeal	Finland	37.6523	0.1836	9	142	317	0.0000	0.0000	0.2233	NE	M
Flour Milling Grade	wholemeal	Switzerlan d	16.2077	0.1205	141	393	317	0.0001	0.0004	1.2642	PE	M
Flour Milling Grade	wholemeal	Germany	15.4812	0.1177	66	158	317	0.0001	0.0005	1.4719	PE	M
Flour Milling Grade	wholemeal	Italy	5.8948	0.0726	13	81	317	0.0152	0.0527	0.5655	NE	M
Flour Organic	yes	Finland	16.4732	0.1225	64	142	669	0.0000	0.0003	0.7397	NE	M
Flour Organic	yes	Italy	10.7441	0.0989	35	81	669	0.0010	0.0054	0.7092	NE	M
Flour Organic	yes	Romania	10.5630	0.0981	17	46	669	0.0012	0.0055	0.6066	NE	M
Flour Organic	yes	Germany	19.7720	0.1342	122	158	669	0.0000	0.0001	1.2673	PE	M
Flour Organic	yes	Austria	7.3368	0.0817	23	26	669	0.0068	0.0250	1.4519	PE	M
Flour Type (Grain)	other	Germany	7.7723	0.0848	9	156	141	0.0053	0.0214	0.4423	NE	M
Flour Type (Grain)	other	Denmark	20.7979	0.1387	9	17	141	0.0000	0.0000	4.0588	PE	M
Flour Type (Grain)	other	Sweden	9.9093	0.0957	9	25	141	0.0016	0.0076	2.7600	PE	M
Flour Type (Grain)	other	United Kingdom	4.5618	0.0650	5	14	141	0.0327	0.1083	2.7381	PE	M
Flour Type (Grain)	rye	Germany	49.2316	0.2134	79	156	294	0.0000	0.0000	1.8620	PE	M
Flour Type (Grain)	rye	Austria	35.8923	0.1822	21	26	294	0.0000	0.0000	2.9698	PE	M
Flour Type (Grain)	rye	Finland	7.6379	0.0841	24	140	294	0.0057	0.0223	0.6303	NE	M
Flour Type (Grain)	rye	Romania	6.3317	0.0765	4	43	294	0.0119	0.0425	0.3420	NE	M
Flour Type (Grain)	spelt	Switzerlan d	18.7321	0.1316	33	383	51	0.0000	0.0001	1.8263	PE	U
Flour Type (Grain)	wheat	Italy	35.3790	0.1809	70	80	595	0.0000	0.0000	1.5897	PE	M
Flour Type (Grain)	wheat	Finland	21.4634	0.1409	103	140	595	0.0000	0.0000	1.3367	PE	M

Flour Type (Grain)	wheat	Germany	19.4771	0.1342	60	156	595	0.0000	0.0001	0.6988	NE	M
Flour Type (Grain)	wheat	Austria	12.3632	0.1069	5	26	595	0.0004	0.0025	0.3494	NE	M
Flour Type (Grain)	wheat	Switzerland	8.8784	0.0906	187	383	595	0.0029	0.0128	0.8871	NE	M
Flour Type (Grain)	wheat	Romania	7.6346	0.0840	33	43	595	0.0057	0.0223	1.3943	PE	M
Water Source	bottled water	Switzerland	7.3664	0.0818	8	391	49	0.0066	0.0250	0.4601	NE	M
Water Source	bottled water	France	43.8491	0.1995	8	23	49	0.0000	0.0000	7.8225	PE	M
Water Source	bottled water	Romania	15.8865	0.1201	8	46	49	0.0001	0.0004	3.9113	PE	M
Water Source	bottled water	Italy	14.9246	0.1164	11	81	49	0.0001	0.0007	3.0542	PE	M
Water Source	spring water	Romania	47.6393	0.2079	5	46	9	0.0000	0.0000	13.3092	PE	U
Water Source	tap water	Romania	43.6804	0.1991	22	46	925	0.0000	0.0000	0.5698	NE	M
Water Source	tap water	France	20.0686	0.1349	11	23	925	0.0000	0.0001	0.5698	NE	M
Water Source	tap water	Switzerland	7.8132	0.0842	345	391	925	0.0052	0.0213	1.0512	PE	M
Water Source	tap water	Austria	3.9483	0.0599	26	26	925	0.0469	0.1488	1.1914	PE	M
Water Source	tap water	Finland	14.0503	0.1129	135	142	925	0.0002	0.0010	1.1326	PE	M
Water Source	tap water	Belgium	8.2023	0.0863	75	102	925	0.0042	0.0182	0.8760	NE	M
Water Source	tap water (descaled)	Germany	9.7766	0.0942	22	158	83	0.0018	0.0080	1.8487	PE	U
Water Source	tap water (filtered)	Romania	41.2981	0.1936	9	46	32	0.0000	0.0000	6.7378	PE	M
Water Source	tap water (filtered)	Belgium	11.7526	0.1033	9	102	32	0.0006	0.0033	3.0386	PE	M
Water Source	tap water (filtered)	France	5.2865	0.0693	3	23	32	0.0215	0.0734	4.4918	PE	M
Yeast Usage Prior	yes	Finland	138.6970	0.3548	26	142	696	0.0000	0.0000	0.2899	NE	M
Yeast Usage Prior	yes	Romania	26.7130	0.1557	12	46	696	0.0000	0.0000	0.4130	NE	M
Yeast Usage Prior	yes	Germany	38.2563	0.1863	135	158	696	0.0000	0.0000	1.3528	PE	M
Yeast Usage Prior	yes	Switzerland	32.3141	0.1712	291	391	696	0.0000	0.0000	1.1784	PE	M
Yeast Usage Prior	yes	Austria	4.3669	0.0629	22	26	696	0.0366	0.1179	1.3397	PE	M

Supplementary Table 9. Chi-square enrichment test results for sourdough baking motivations and perceived health benefits across countries (related to Figure 7B). *p*-values were corrected using the Benjamini–Hochberg false discovery rate (BH-FDR). Indicator codes: PE = positive enrichment, NE = negative enrichment, M = multiple indicators, U = unique indicator.

Feature	*Category*	*Cluster*	*Chi2 Statistic*	*Cramér's V*	*Cluster Count with Category*	*Cluster Total*	*Category Total*	*Raw p-value*	*Corrected p-value*	*Enrichment Score*	*Interpretation*	*Indicator*
Baking Motivation: Enjoy Baking	yes	Belgium	20.0601	0.1340	59	113	791	0.0000	0.0005	0.7373	NE	M
Baking Motivation: Enjoy Baking	yes	Germany	7.6165	0.0826	127	158	791	0.0058	0.0606	1.1351	PE	M
Baking Motivation: Enjoy Baking	yes	Finland	5.5681	0.0706	113	142	791	0.0183	0.1338	1.1237	PE	M
Baking Motivation: Health	yes	Italy	31.0100	0.1666	17	81	574	0.0000	0.0000	0.4084	NE	M
Baking Motivation: Health	yes	Romania	12.7701	0.1069	36	46	574	0.0004	0.0130	1.5230	PE	M
Baking Motivation: Health	yes	Germany	6.0409	0.0735	96	158	574	0.0140	0.1114	1.1824	PE	M
Baking Motivation: Intolerance/Allergy	yes	Finland	4.0948	0.0605	5	142	15	0.0430	0.2252	2.6221	PE	M
Baking Motivation: Lower Cost	yes	Switzerland	12.5328	0.1059	36	393	160	0.0004	0.0130	0.6395	NE	M
Baking Motivation: Lower Cost	yes	Hungary	9.8135	0.0937	6	12	160	0.0017	0.0385	3.4906	PE	M
Baking Motivation: Lower Cost	yes	Slovenia	8.3928	0.0867	6	13	160	0.0038	0.0503	3.2221	PE	M
Baking Motivation: Lower Cost	yes	Sweden	8.0671	0.0850	9	25	160	0.0045	0.0547	2.5133	PE	M
Baking Motivation: Lower Cost	yes	Finland	6.7859	0.0779	31	142	160	0.0092	0.0876	1.5241	PE	M
Baking Motivation: Other Reasons	yes	Italy	7.6495	0.0828	6	81	26	0.0057	0.0606	3.1823	PE	M
Baking Motivation: Sustainability	yes	Switzerland	9.3261	0.0914	14	393	20	0.0023	0.0431	1.9896	PE	M
Baking Motivation: Taste	yes	Romania	9.0583	0.0901	28	46	888	0.0026	0.0450	0.7657	NE	M
Baking Motivation: Taste	yes	Italy	6.4603	0.0761	55	81	888	0.0110	0.0935	0.8541	NE	M
Baking Motivation: Taste	yes	Czechia	6.3181	0.0752	3	8	888	0.0120	0.0982	0.4717	NE	M
Baking Motivation: Taste	yes	Finland	5.5745	0.0706	124	142	888	0.0182	0.1338	1.0984	PE	M
Baking Motivation: Taste	yes	Belgium	5.2624	0.0686	80	113	888	0.0218	0.1499	0.8905	NE	M
Baking Motivation: Tradition	yes	Czechia	7.0315	0.0793	3	8	80	0.0080	0.0792	5.2359	PE	M

Baking Motivation: Tradition	yes	Finland	4.8617	0.0660	17	142	80	0.0275	0.1705	1.6716	PE	M
Baking Motivation: Tradition	yes	Sweden	4.5176	0.0636	5	25	80	0.0335	0.2013	2.7925	PE	M
Perceived Benefit: Better Digestibility	yes	Romania	8.6579	0.0880	32	46	528	0.0033	0.0470	1.4717	PE	M
Perceived Benefit: Better Digestibility	yes	Denmark	4.9304	0.0664	3	17	528	0.0264	0.1705	0.3733	NE	M
Perceived Benefit: Better Digestibility	yes	Italy	4.1245	0.0608	29	81	528	0.0423	0.2252	0.7574	NE	M
Perceived Benefit: Gut Health	yes	Germany	13.7568	0.1110	5	158	140	0.0002	0.0111	0.2525	NE	M
Perceived Benefit: Gut Health	yes	Romania	9.3801	0.0916	13	46	140	0.0022	0.0431	2.2548	PE	M
Perceived Benefit: Gut Health	yes	United Kingdom	4.9729	0.0667	5	14	140	0.0257	0.1705	2.8495	PE	M
Perceived Benefit: Gut Health	yes	Sweden	4.2302	0.0615	7	25	140	0.0397	0.2164	2.2340	PE	M
Perceived Benefit: Healthier Ingredients	yes	Switzerlan d	11.0500	0.0995	25	393	119	0.0009	0.0237	0.5971	NE	M
Perceived Benefit: Healthier Ingredients	yes	Germany	8.8130	0.0888	28	158	119	0.0030	0.0456	1.6634	PE	M
Perceived Benefit: Healthier Ingredients	yes	France	7.6486	0.0827	7	23	119	0.0057	0.0606	2.8568	PE	M
Perceived Benefit: Increased Satiety	yes	France	4.4023	0.0628	3	23	36	0.0359	0.2083	4.0471	PE	M
Perceived Benefit: Intolerance/Allergy	yes	Switzerlan d	4.3247	0.0622	36	393	77	0.0376	0.2130	1.3288	PE	M
Perceived Benefit: Mental Health	yes	Denmark	4.9009	0.0662	3	17	46	0.0268	0.1705	4.2852	PE	M
Perceived Benefit: Nutrient Availability	yes	Sweden	8.9169	0.0893	9	25	153	0.0028	0.0456	2.6282	PE	M
Perceived Benefit: Nutrient Availability	yes	Austria	8.1248	0.0853	9	26	153	0.0044	0.0547	2.5271	PE	M
Perceived Benefit: Nutrient Availability	yes	Italy	6.4952	0.0763	3	81	153	0.0108	0.0935	0.2704	NE	M
Perceived Benefit: Nutrient Availability	yes	Switzerlan d	5.9016	0.0727	40	393	153	0.0151	0.1171	0.7431	NE	M
Perceived Benefit: Stable Glucose Levels	yes	Romania	21.9975	0.1403	9	46	50	0.0000	0.0003	4.3709	PE	M
Perceived Health Benefits	yes	Italy	12.4676	0.1083	55	79	895	0.0004	0.0130	0.8269	NE	M
Perceived Health Benefits	yes	Germany	6.5616	0.0786	116	151	895	0.0104	0.0935	0.9124	NE	M
Perceived Health Benefits	yes	Romania	3.8853	0.0605	44	46	895	0.0487	0.2501	1.1361	PE	M
Perceived Health Benefits	yes	Austria	3.8594	0.0603	26	26	895	0.0495	0.2516	1.1877	PE	M
Self-Reported Sourdough Skill (Years)	<0.5	Germany	11.1784	0.1021	3	153	104	0.0008	0.0237	0.2023	NE	M

Self-Reported Sourdough Skill (Years)	<0.5	Switzerland	5.2984	0.0703	48	380	104	0.0213	0.1499	1.3032	PE	M
Self-Reported Sourdough Skill (Years)	>5	Germany	21.3039	0.1409	66	153	294	0.0000	0.0003	1.5744	PE	M
Self-Reported Sourdough Skill (Years)	>5	Sweden	9.1046	0.0921	14	25	294	0.0025	0.0450	2.0438	PE	M
Self-Reported Sourdough Skill (Years)	>5	Switzerland	7.8843	0.0857	84	380	294	0.0050	0.0592	0.8068	NE	M
Self-Reported Sourdough Skill (Years)	>5	Finland	5.3098	0.0703	27	142	294	0.0212	0.1499	0.6939	NE	M
Self-Reported Sourdough Skill (Years)	>5	Italy	3.9015	0.0603	30	80	294	0.0482	0.2501	1.3686	PE	M
Self-Reported Sourdough Skill (Years)	0.5 to 2	Norway	10.5138	0.0990	3	3	167	0.0012	0.0301	6.4251	PE	M
Self-Reported Sourdough Skill (Years)	0.5 to 2	Germany	10.2803	0.0979	10	153	167	0.0013	0.0326	0.4199	NE	M
Self-Reported Sourdough Skill (Years)	0.5 to 2	Netherlands	4.5965	0.0655	8	24	167	0.0320	0.1966	2.1417	PE	M
Self-Reported Sourdough Skill (Years)	2 to 5	Finland	22.3861	0.1444	83	142	429	0.0000	0.0003	1.4619	PE	M
Self-Reported Sourdough Skill (Years)	2 to 5	Sweden	7.2004	0.0819	3	25	429	0.0073	0.0748	0.3001	NE	M
Self-Reported Sourdough Skill (Years)	2 to 5	Romania	4.4952	0.0647	11	46	429	0.0340	0.2017	0.5981	NE	M
Self-Reported Sourdough Skill (Years)	professional	Czechia	13.3896	0.1117	3	5	79	0.0003	0.0123	8.1494	PE	M
Self-Reported Sourdough Skill (Years)	professional	Switzerland	6.6145	0.0785	39	380	79	0.0101	0.0935	1.3940	PE	M

Supplementary Table 10. Chi-square enrichment test results for sourdough storage and home/bakery environments across countries. p-values were corrected using the Benjamini–Hochberg false discovery rate (BH-FDR). Indicator codes: PE = positive enrichment, NE = negative enrichment, M = multiple indicators, U = unique indicator.

Feature	*Category*	*Cluster*	*Chi2 Statistic*	*Cramér's V*	*Cluster Count with Category*	*Cluster Total*	*Category Total*	*Raw p-value*	*Corrected p-value*	*Enrichment Score*	*Interpretation*	*Indicator*
Dough Storage Container	glass jar	United Kingdom	5.2668	0.0691	8	14	919	0.0217	0.1261	0.6852	NE	M
Dough Storage Container	glass jar	Finland	4.8135	0.0661	128	142	919	0.0282	0.1551	1.0809	PE	M
Dough Storage Container	other	United Kingdom	19.3020	0.1323	3	14	21	0.0000	0.0004	11.2449	PE	M
Dough Storage Container	plastic container	Finland	6.9390	0.0794	10	142	162	0.0084	0.0756	0.4790	NE	U
Dough Storage Cover	closed lid	Finland	20.4713	0.1363	102	142	593	0.0000	0.0003	1.3349	PE	M
Dough Storage Cover	closed lid	Switzerland	15.2300	0.1176	179	391	593	0.0001	0.0020	0.8508	NE	M

Dough Storage Cover	closed lid	Germany	12.4660	0.1064	106	158	593	0.0004	0.0068	1.2467	PE	M
Dough Storage Cover	fabric	Italy	22.3307	0.1424	9	81	28	0.0000	0.0001	4.3730	PE	M
Dough Storage Cover	fabric	Netherlands	6.1456	0.0747	3	24	28	0.0132	0.0974	4.9196	PE	M
Dough Storage Cover	loose lid	Finland	13.4132	0.1103	33	142	413	0.0002	0.0044	0.6201	NE	M
Dough Storage Cover	loose lid	Switzerland	11.4041	0.1017	173	391	413	0.0007	0.0117	1.1806	PE	M
Dough Storage Cover	loose lid	Denmark	6.7071	0.0780	12	17	413	0.0096	0.0845	1.8835	PE	M
Dough Storage Cover	loose lid	Italy	4.4607	0.0636	21	81	413	0.0347	0.1727	0.6918	NE	M
Dough Storage Cover	other	Italy	4.2603	0.0622	5	81	25	0.0390	0.1885	2.7210	PE	M
Dough Storage Cover	plastic wrap	Switzerland	7.1833	0.0807	24	391	43	0.0074	0.0672	1.5731	PE	M
Dough Storage Cover	plastic wrap	Austria	6.4896	0.0767	4	26	43	0.0109	0.0922	3.9428	PE	M
Dough Storage Cover	plastic wrap	Italy	3.9628	0.0600	7	81	43	0.0465	0.2029	2.2148	PE	M
Feeding Storage Location	dough corner in bakery	Germany	18.0757	0.1281	5	158	6	0.0000	0.0006	5.8122	PE	U
Feeding Storage Location	fridge/cool place	Italy	14.1107	0.1132	18	81	107	0.0002	0.0031	2.2887	PE	M
Feeding Storage Location	fridge/cool place	Germany	5.1817	0.0686	7	158	107	0.0228	0.1282	0.4563	NE	M
Feeding Storage Location	kitchen	Germany	31.5897	0.1693	66	158	685	0.0000	0.0000	0.6720	NE	M
Feeding Storage Location	kitchen	Belgium	7.8789	0.0846	77	102	685	0.0050	0.0493	1.2145	PE	M
Feeding Storage Location	kitchen	Hungary	5.8486	0.0729	12	12	685	0.0156	0.1038	1.6088	PE	M
Feeding Storage Location	other	Italy	5.9005	0.0732	6	81	29	0.0151	0.1022	2.8148	PE	M
Feeding Storage Location	other	Finland	4.4678	0.0637	8	142	29	0.0345	0.1727	2.1408	PE	M
Feeding Storage Location	proofing chamber	Germany	99.3663	0.3003	53	158	117	0.0000	0.0000	3.1595	PE	M
Feeding Storage Location	proofing chamber	Finland	9.5285	0.0930	4	142	117	0.0020	0.0270	0.2653	NE	M
Feeding Storage Location	proofing chamber	Spain	4.6758	0.0651	3	7	117	0.0306	0.1635	4.0366	PE	M
Feeding Storage Location	proofing chamber	Belgium	4.5609	0.0643	4	102	117	0.0327	0.1662	0.3694	NE	M
Feeding Storage Location	warm place	Finland	4.3619	0.0629	29	142	158	0.0368	0.1812	1.4244	PE	U

Fermented Acidic Beverages	yes	United Kingdom	8.5751	0.0876	6	14	146	0.0034	0.0373	3.2789	PE	M
Fermented Acidic Beverages	yes	Belgium	5.1778	0.0681	23	113	146	0.0229	0.1282	1.5572	PE	M
Fermented Acidic Beverages	yes	Finland	4.6133	0.0643	10	142	146	0.0317	0.1635	0.5388	NE	M
Fermented Alcoholic Beverages	yes	Slovenia	5.4213	0.0697	3	13	57	0.0199	0.1182	4.5223	PE	M
Fermented Alcoholic Beverages	yes	Sweden	4.1801	0.0612	4	25	57	0.0409	0.1920	3.1354	PE	M
Fermented Dairy Products	yes	Germany	20.1523	0.1343	54	158	229	0.0000	0.0003	1.6671	PE	M
Fermented Dairy Products	yes	Finland	19.0396	0.1306	9	142	229	0.0000	0.0004	0.3092	NE	M
Fermented Dairy Products	yes	France	16.5063	0.1216	13	23	229	0.0000	0.0011	2.7570	PE	M
Fermented Dairy Products	yes	Austria	9.1967	0.0907	12	26	229	0.0024	0.0292	2.2513	PE	M
Fermented Vegetables/Fruits	yes	Finland	14.8221	0.1152	23	142	340	0.0001	0.0022	0.5321	NE	M
Fermented Vegetables/Fruits	yes	Hungary	9.3482	0.0915	9	12	340	0.0022	0.0282	2.4640	PE	M
Fermented Vegetables/Fruits	yes	United Kingdom	6.1376	0.0741	9	14	340	0.0132	0.0974	2.1120	PE	M
Fermented Vegetables/Fruits	yes	Austria	5.8030	0.0721	14	26	340	0.0160	0.1038	1.7690	PE	M
Fermented Vegetables/Fruits	yes	Norway	3.9748	0.0597	3	3	340	0.0462	0.2029	3.2853	PE	M
Other Fermentation Types	yes	Italy	6.1799	0.0744	8	81	45	0.0129	0.0974	2.4516	PE	M
Other Fermentation Types	yes	Switzerlan d	4.0718	0.0604	9	393	45	0.0436	0.1985	0.5684	NE	M
Other Fermentations	yes	Finland	35.7845	0.1819	35	142	523	0.0000	0.0000	0.5095	NE	M
Other Fermentations	yes	Hungary	7.4356	0.0829	11	12	523	0.0064	0.0595	1.8947	PE	M
Other Fermentations	yes	Sweden	4.7894	0.0666	18	25	523	0.0286	0.1551	1.4882	PE	M
Other Fermentations	yes	Germany	4.0156	0.0609	86	153	523	0.0451	0.2002	1.1618	PE	M
Ownership of Animals (No Hair)	yes	Sweden	7.9960	0.0846	3	25	23	0.0047	0.0472	5.8278	PE	M
Ownership of Cat	yes	Belgium	16.0826	0.1200	41	113	237	0.0001	0.0013	1.7101	PE	M
Ownership of Cat	yes	Germany	7.4804	0.0818	20	158	237	0.0062	0.0591	0.5966	NE	M
Ownership of Cat	yes	France	5.6684	0.0712	10	23	237	0.0173	0.1051	2.0492	PE	M
Ownership of Dog	yes	Germany	5.9372	0.0729	15	158	184	0.0148	0.1020	0.5763	NE	M

Ownership of Dog	yes	Spain	5.7548	0.0718	4	7	184	0.0164	0.1039	3.4689	PE	M
Pet Ownership	yes	Belgium	19.3977	0.1327	57	102	391	0.0000	0.0004	1.5736	PE	M
Pet Ownership	yes	Germany	10.0073	0.0953	38	158	391	0.0016	0.0226	0.6772	NE	M
Pet Ownership	yes	Spain	5.7032	0.0720	6	7	391	0.0169	0.1051	2.4136	PE	M
Pet Ownership	yes	Netherlands	4.6069	0.0647	14	24	391	0.0318	0.1635	1.6426	PE	M
Presence of Kids	yes	Belgium	8.6006	0.0890	50	101	388	0.0034	0.0373	1.3869	PE	M
Presence of Kids	yes	Germany	5.9466	0.0740	42	157	388	0.0147	0.1020	0.7495	NE	M
Presence of Kids	yes	Czechia	4.0608	0.0611	5	6	388	0.0439	0.1985	2.3346	PE	M
Presence of Plants	yes	Switzerland	26.4896	0.1560	157	387	557	0.0000	0.0000	0.7924	NE	M
Presence of Plants	yes	Sweden	9.7183	0.0945	21	25	557	0.0018	0.0250	1.6408	PE	M
Presence of Plants	yes	Belgium	8.3105	0.0874	66	101	557	0.0039	0.0413	1.2764	PE	M
Presence of Plants	yes	Germany	6.1794	0.0754	65	156	557	0.0129	0.0974	0.8139	NE	M
Presence of Plants	yes	Finland	4.2444	0.0625	83	139	557	0.0394	0.1885	1.1664	PE	M
Storage After Feeding	fridge	Finland	40.4009	0.1933	84	142	860	0.0000	0.0000	0.7436	NE	M
Storage After Feeding	fridge	Switzerland	18.6984	0.1315	331	381	860	0.0000	0.0004	1.0920	PE	M
Storage After Feeding	fridge	Germany	6.4952	0.0775	134	153	860	0.0108	0.0922	1.1009	PE	M
Storage After Feeding	keep at rt	Finland	34.9822	0.1799	53	142	204	0.0000	0.0000	1.9778	PE	M
Storage After Feeding	keep at rt	Switzerland	13.2838	0.1109	49	381	204	0.0003	0.0046	0.6815	NE	M
Storage After Feeding	keep at rt	Germany	6.4325	0.0771	17	153	204	0.0112	0.0936	0.5888	NE	M
Storage After Feeding	keep at rt	Czechia	6.1369	0.0753	4	6	204	0.0132	0.0974	3.5327	PE	M

Supplementary list 1. Deduplicated and curated list of the main questions and interests obtained from the participants to the co-design workshops across Europe.

- What are the health effects of using sourdough?
- Does the microbial composition of a sourdough change over time?
- Why does sourdough sometimes go bad, which microbes can do it?
- Does the method of storing a sourdough influence it?
- Is there a difference between sourdoughs made with additive-free flour and normal flour?
- Can you change or regulate the taste and function of a sourdough?
- Do different sourdoughs have different microbes?
- Does a change in cereal flour type have an effect on the sourdough?
- Is filtered water okay for use in a sourdough?
- What is the optimal feeding frequency?
- How long can you leave your sourdough without feeding?
- What are the influences of the addition of different ingredients?
- How much does a starter vary between different timepoints? When is it stable?
- Which microorganisms are the most useful in my sourdough and can I selectively enrich them in some way?
- Does the air quality/ pollution influence a starter more than the general geolocation?
- Most important influences on the sourdough?
- Which microorganisms are present and is there a link with the conditions?
- Does my sourdough contain rare microorganisms?
- What is the link between a sourdough and the sensorial properties of the bread?
- How acidic is my sourdough? Which compounds are present? Are there unique ones, absences, health benefits related to some,...
- Is a professional sourdough starter different? Is it easier to maintain?
- Is it safe to eat a sourdough starter without baking it?
- Is it possible to use gut-bacteria to inoculate a sourdough starter?
- Is the microbial activity of the sourdough affecting the dough behaviour and the bread?
- Can you use gluten-free flour to bake sourdough bread?
- Does the taste of the bread change depending on the acids that are produced in the sourdough?
